# Immune modulation underpins the anti‐cancer activity of HDAC inhibitors

**DOI:** 10.1002/1878-0261.12953

**Published:** 2021-05-01

**Authors:** Wiktoria Blaszczak, Geng Liu, Hong Zhu, Wojciech Barczak, Amit Shrestha, Gulsah Albayrak, Shunsheng Zheng, David Kerr, Anastasia Samsonova, Nicholas B. La Thangue

**Affiliations:** ^1^ Celleron Therapeutics Ltd Oxford UK; ^2^ Laboratory of Cancer Biology Department of Oncology University of Oxford UK; ^3^ Department of Medical Oncology Cancer Center West China Hospital Sichuan University Chengdu China; ^4^ Nuffield Division of Clinical Laboratory Sciences University of Oxford UK; ^5^ Centre for Computational Biology Peter the Great Saint Petersburg Polytechnic University Russia; ^6^ Centre for Genome Bioinformatics St. Petersburg State University Russia

**Keywords:** checkpoints inhibitors, HDAC inhibitors, immunotherapy, tumour microenvironment

## Abstract

Aberrant protein acetylation is strongly linked to tumorigenesis, and modulating acetylation through targeting histone deacetylase (HDAC) with small‐molecule inhibitors has been the focus of clinical trials. However, clinical success on solid tumours, such as colorectal cancer (CRC), has been limited, in part because the cancer‐relevant mechanisms through which HDAC inhibitors act remain largely unknown. Here, we have explored, at the genome‐wide expression level, the effects of a novel HDAC inhibitor CXD101. In human CRC cell lines, a diverse set of differentially expressed genes were up‐ and downregulated upon CXD101 treatment. Functional profiling of the expression data highlighted immune‐relevant concepts related to antigen processing and natural killer cell‐mediated cytotoxicity. Similar profiles were apparent when gene expression was investigated in murine colon26 CRC cells treated with CXD101. Significantly, these changes were also apparent in syngeneic colon26 tumours growing *in vivo*. The ability of CXD101 to affect immune‐relevant gene expression coincided with changes in the tumour microenvironment (TME), especially in the subgroups of CD4 and CD8 tumour‐infiltrating T lymphocytes. The altered TME reflected enhanced antitumour activity when CXD101 was combined with immune checkpoint inhibitors (ICIs), such as anti‐PD‐1 and anti‐CTLA4. The ability of CXD101 to reinstate immune‐relevant gene expression in the TME and act together with ICIs provides a powerful rationale for exploring the combination therapy in human cancers.

## Graphical abstract

CXD101 is a novel histone deacetylase inhibitor with potent antitumour activity. We find that CXD101 reinstates immune‐relevant gene expression in tumours, which includes major histocompatibility complex class I and class II genes. This enables CXD101 to enhance the activity of immune checkpoint therapies, such as anti‐PD‐1, on tumours that would otherwise be poorly responsive and coincides with increased T lymphocyte infiltration into the tumour microenvironment.

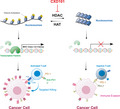

**Abbreviations**
AP: antigen processing and presentation
APCs: antigen‐presenting cells
CRC: colorectal cancer
DEGs: differentially expressed genes
GTEx: Genotype‐Tissue Expression
HATs: histone acetyltransferases
HDAC: histone deacetylase
ICIs: immune checkpoint inhibitors
IHC: immunohistochemistry
MHC: major histocompatibility complex
MSS/MSI: microsatellite stable/instable
NK: natural killer cell‐mediated cytotoxicity
TME: tumour microenvironment
Tregs: T regulatory cells

## 1 Introduction

Lysine acetylation is regulated by two groups of enzymes: Histone acetyltransferases (HATs) mediate the acetylation event [1], and histone deacetylases (HDACs) regulate the deacetylation event [2]. Lysine acetylation occurs on many proteins and therefore influences pathways with diverse functional roles [3]. Significantly, aberrant protein acetylation is recognised to take on an important role in driving the malignant phenotype [4]; thus, deregulation of HDAC activity occurs in different types of cancer and HDAC as a cancer drug target has been validated in many preclinical models [5]. Therapeutically, however, clinical success in human disease has been surprisingly limited [6]; most clinical activity has been observed in haematological malignancies [7] where recent HDAC inhibitor drug approvals include panobinostat for multiple myeloma and chidamide for T‐cell lymphoma [8]. Generally speaking, HDAC inhibitors have met with limited success in solid cancer clinical trials [7]. For example, in clinical trials on colorectal cancer (CRC), negligible activity was observed in treated individuals [9]. This may reflect our limited insights into the key molecular mechanisms and cancer‐relevant pathways upon which HDAC inhibitors act. Having this information at hand could allow for a more scientifically driven and rational clinical development plan.

CXD101 is a promising second‐generation inhibitor with selective activity towards class 1 HDAC subunits [10]. It is a potent antiproliferative agent, which in human clinical studies demonstrated a favourable safety profile [10]. In addition, encouraging durable clinical activity was seen in a phase I clinical trial in patients with T‐cell lymphoma, follicular lymphoma and Hodgkin lymphoma (including postallogenic stem cell transplantation), with tumour reduction evident in 63% of patients [10]. Although efficacious in haematological malignancy, we wanted to develop a scientific rationale for deploying CXD101 in the solid cancer setting [11].

With this objective in mind, we have sought to explore the mechanisms through which CXD101 acts. By performing a genome‐wide expression analysis on human CRC cells, we identified a diverse set of differentially expressed genes (DEGs) upon treatment with CXD101. Functional profiling of the gene expression data highlighted biologically enriched concepts related to immune recognition, specifically antigen presentation (AP) and natural killer (NK) cell activity. Similar concepts were apparent in gene expression data derived from the murine CRC cell line colon26 treated with CXD101 *in vitro* and in colon26 syngeneic tumours growing *in vivo*. The enriched immune recognition concepts reflected changes in the tumour microenvironment (TME), where a marked effect on the population of tumour‐infiltrating lymphocytes and other immune relevant cells was observed upon treatment. These results led us to test the therapeutic impact of CXD101 in combination with agents that act through the immune system, such as the immune checkpoint inhibitors (ICIs) anti‐PD‐1 and anti‐CTLA4 [12]. Under conditions where there was minimal effect of the ICI monotherapy, enhanced antitumour activity was observed in the CXD101 combination treatment, suggesting that the gene expression changes and the subsequent impact on antigen presentation in the TME act to enhance the antitumour effects of ICIs. Our results have important implications for understanding the mechanisms, which underpin HDAC inhibitor‐based therapies and provide a powerful rationale for testing the combined effect of CXD101 with ICIs in human solid malignancies.

## 2 Materials and methods

### 2.1 Cell culture and compound treatment

Human colorectal adenocarcinoma SW620 (ATCC^®^ CCL‐227; RRID:CVCL_0547) and HCT116 (ATCC^®^ CCL‐247; RRID:CVCL_0291), human breast cancer MCF7 (ATCC^®^ HTB‐22; RRID:CVCL_0031), human lung cancer A549 (ATCC^®^ CCL‐185; RRID:CVCL_JK07) and the mouse colon carcinoma cell line colon26 (ATCC^®^ CRL‐2639; RRID:CVCL_7255) were obtained from ATCC (Manassas, VA, USA). Human cell lines were cultured in Dulbecco's modified Eagle medium (Sigma‐Aldrich, St. Louis, MO, USA) supplemented with 10% FBS (Labtech, Heathfield, UK) and 1% penicillin/streptomycin (Gibco, Life Technologies, Carlsbad, CA, USA), while colon26 cells in RPMI (Sigma‐Aldrich). All cell lines were tested for mycoplasma contamination before use. CXD101 was used as described [10].

### 2.2 MTT assay

Cells were seeded onto 96‐well plates overnight and the next day dosed with CXD101 and incubated for 72 or 120 h. Next, 100 µL of thiazolyl blue tetrazolium bromide (MTT; Sigma‐Aldrich) was added into a well (final concentration 5 µm) and incubated for 2 h at 37 °C. After that medium was discarded and formazan crystals were dissolved in 100 µL DMSO (VWR International, Radnor, PA, USA) by shaking for 15 min. Absorbance was read by Omega FLUOstar plate reader (BMG Labtech Ltd, Ortenberg, Germany) at the 584 nm wavelength. Data were analysed, and IC_50_ doses were calculated in graphpad prism 8 (GraphPad Software, San Diego, CA, USA; RRID:SCR_002798).

### 2.3 RNA extraction library preparation and RNA‐seq analysis

SW620 and colon26 cells were treated as described with CXD101 or DMSO as a negative control. Total RNA (triplicates unless otherwise stated) was isolated using Direct‐zol RNA MiniPrep Kit (Zymo Research, Irvine, CA, USA) according to the manufacturer's instructions. RNA sequencing was performed by BGI Genomics (Beijing, China). Briefly, an Agilent 2100 Bioanalyzer (Agilent RNA 6000 Nano Kit; Santa Clara, CA, USA) was used for RNA sample quality control purposes (RNA concentration, RIN value, 28S/18S and the fragment length distribution). mRNAs were isolated from total RNA using the oligo(dT) method. Then, the mRNAs were fragmented, and first‐strand/second‐strand complementary DNAs (cDNAs) were synthesised. cDNA fragments were purified and resolved with EB buffer for end reparation and single nucleotide A (adenine) addition. Subsequently, the cDNA fragments were linked with adapters. Those cDNA fragments with suitable size were selected for the PCR amplification. Agilent 2100 Bioanalyzer and ABI StepOnePlus Real‐Time PCR System were used in quantification and qualification of those libraries. The RNA sequencing was carried out using Illumina HiSeq Platform (SW620) or BGI500 platform (colon26 *in vitro* and *in vivo*).

### 2.4 RT‐qPCR

RNA was isolated from cells using TRIzol (Thermo Fisher Scientific, Waltham, MA, USA) or the Direct‐zol RNA MiniPrep Kit (Zymo Research) according to the manufacturer's instructions. One microgram of total RNA was used for cDNA synthesis. Reverse transcription with oligo(dT)20 primer (Invitrogen, Carlsbad, CA, USA) was performed using SuperScript III Reverse Transcriptase (Invitrogen) as per the manufacturer's instructions. Quantitative reverse transcription PCR (qRT‐PCR) was carried out in technical triplicate using the indicated primer pairs and the Brilliant III Ultra‐Fast SYBR^®^ Green qPCR Master Mix (Agilent) on an AriaMX Real‐Time qPCR Instrument (Agilent). Results were expressed as average (mean) fold change compared with control treatments using the ΔΔ*C*_t_ method from three biological repeat experiments. Glyceraldehyde phosphate dehydrogenase primer sets were used as an internal calibrator. Error bars represent SE unless otherwise indicated.

### 2.5 Genome‐wide expression analysis in human and mouse cell lines and tissues

FASTQ files for CXD101 and DMSO‐treated SW620 and colon26 cells were generated. Sequencing reads were trimmed to remove adapters and low‐quality bases with trimgalore v.0.4.3 (http://www.bioinformatics.babraham.ac.uk/projects/trim_galore/) (RRID:SCR_011847). Likewise, FASTQ files for experiments addressing CXD101 treatment in mice were generated in four biological replicates and reads were trimmed as described above. The expression data for all three experiments have been further processed as follows; the trimmed reads were aligned to the human and mouse reference genomes (builds hg19 and mm10, respectively) with star aligner v.2.7 (RRID:SCR_015899) [13] with two mismatches allowed. Differential gene expression analysis was done with deseq2 r bioconductor package (v.1.22) (RRID:SCR_000154; RRID:SCR_006442) [14], using read count data provided by the aligner. Genes were considered differentially expressed if the adjusted *P*‐value, calculated using the Benjamini–Hochberg method in order to minimise the false discovery rate, was less than 0.01. We further filtered significantly DEG sets to select for genes expressed at high levels using twofold change in absolute expression levels for both human and mouse data sets.

Gene expression data have been deposited in NCBI's Gene Expression Omnibus and are accessible through GEO Series (RRID:SCR_005012) Accession Number GSE158164.

### 2.6 Gene set enrichment analyses

Genes significantly differentially expressed upon CXD101 treatment of SW620 cells were further subjected to enrichment analyses in the pi [15] and xgr software packages (v.1.12 and v1.1.6, respectively) [16] to reveal signalling and metabolic pathways over‐represented in the DEG sets. For pathway‐based gene enrichment analyses, we used Reactome Knowledge Base [17] pathways and, more specifically, superpathways that correspond to domains of biology such as immune system and signal transduction. *P*‐values for pathway enrichment analyses were calculated using the formula for hypergeometric distribution, reflecting the probability for a pathway to arise by chance. Significantly enriched pathways were identified using a threshold FDR of 0.05.

### 2.7 Parametric GSEA

Parametric gene set enrichment analysis was performed with r pgsea package (v. 1.58) [18] on the collections of curated gensets (c2) derived from the KEGG pathway database (RRID:SCR_012773) available from the Broad Institute's Molecular Signatures Database (MSigDB v. 6.2). The expression matrix used in these analyses was normalised and rlog‐transformed with deseq2 r package. Gene sets with less than 10 genes and more than 10 000 genes were excluded from the analyses. A linear model was applied employing the *limma* package (RRID:SCR_010943) (v.3.44.0) [19] followed by empirical Bayesian analysis to determine concepts associated with significant differences between treated and untreated samples. Differences were considered significant if the adjusted *P*‐value, calculated using the Benjamini–Hochberg method [20] in order to minimise false discovery rate, was less than 0.005.

To perform parametric gene set enrichment analyses in mouse experiments, we used annotations provided by gskb r bioconductor package [21], which contains molecular signature databases for pathway analysis in the mouse. The procedure for discovery of significantly over‐represented biological concepts (i.e. pathways) was the same as above; however, differences were considered significant if the adjusted *P*‐value was less than 0.01.

### 2.8 Functional genomics analysis

For the analysis of ‘antigen processing and presentation’ and ‘natural killer cell‐mediated cytotoxicity’ pathway gene expression levels in human cancers, Xena Browser (University of California, CA, USA) was used (https://xena.ucsc.edu/) [22]. The TCGA TARGET GTEx data set was selected, which contained transcript expression data from TCGA (https://portal.gdc.cancer.gov/; cancer tissue) and Genotype‐Tissue Expression (GTEx; https://gtexportal.org/home/; healthy tissue) samples. For subsequent detailed analysis of microsatellite stability and staging, TCGA colon, stomach and oesophageal cancer data sets collected from cBioPortal (www.cbioportal.org) were used [23, 24]. Data were presented as heat maps generated using morpheus software (Broad Institute, Cambridge, MA, USA).

### 2.9 Genevestigator analysis

For the analysis of ‘antigen processing and presentation’ and ‘natural killer cell‐mediated cytotoxicity’ pathway gene expression levels in different cell lines treated with HDAC inhibitors, the Genevestigator tool (Nebion AG, Zurich, Switzerland) (RRID:SCR_002358) was used. Data from Subramanian *et al*. [25], Kubo *et al*. [26] and Ghandi *et al*. [27] were collected and presented as graphs or as heat maps generated using morpheus software (Broad Institute, Cambridge, MA, USA).

### 2.10 Western blotting

Cell pellets were lysed in radio‐immunoprecipitation assay buffer [50 mm Tris/HCl (pH 8), 150 mm NaCl, 1% Igepal CA‐630, 0.5% sodium deoxycholate, 0.1% SDS, 0.2 mm sodium orthovanadate and protease inhibitor cocktails], for 30 min on ice and centrifuged for another 30 min at maximum speed at 4 °C. Protein concentration was assessed by Bradford assay (Quick Start™ Bradford 1× Dye Reagent; Bio‐Rad Laboratories, Hercules, CA, USA). After gel electrophoresis, proteins were transferred onto the PVDF or nitrocellulose membrane by means of Trans‐Blot^®^ Turbo™ Transfer System (Bio‐Rad Laboratories) and blocked by 1‐h incubation in 5% skimmed milk (Merck Group, Darmstadt, Germany) in PBST at room temperature. The following antibodies were used in immunoblotting: anti‐H3 (ab1791; Abcam, Cambridge, UK; RRID:AB_302613), anti‐H3AcK9 (ab10812; Abcam; RRID:AB_297491), anti‐H3AcK14 (#7627; Cell Signaling, Danvers, MA, USA; RRID:AB_10839410) and anti‐β‐Actin (#3700; Cell Signaling; RRID:AB_2242334), all overnight at 4 °C. Next, membranes were washed and treated with secondary antibody for 1 h at RT. Chemiluminescent signals were detected by LI‐COR C‐Digit (LI‐COR Biosciences, Lincoln, NE, USA), and the data were quantified using imagej software (National Institutes of Health, Bethesda, MD, USA) (RRID:SCR_003070).

### 2.11 Immunohistochemistry

Tumours were harvested at day 14, embedded in paraffin blocks and cut into 5‐µm sections. FFPE slides were washed for 5 min with HistoChoice (Sigma‐Aldrich), followed by two times of 3‐min washing in 100% ethanol, 3 min in 70% ethanol and 5 min in tap water. Next, samples were incubated with antigen retrieval solution (e.g. sodium citrate buffer or Tris/EDTA – depending on used antibody) at 99 °C in water bath for 20 min. After 3× washing with purified water, samples were incubated in freshly made 6% methanol/H_2_O_2_ for 15 min and washed in tap water. In the next steps, slides were washed in 1% PBST for 5 min, blocked in blocking serum solution (VECTASTAIN ABC Kit; Vector Laboratories, Burlingame, CA, USA) for 20 min., washed again in 1% PBST for 5 min and incubated overnight at 4 °C (staining with anti‐H3AcK9 was performed for 8 min at room temperature) with primary antibody: anti‐H3AcK9 (ab10812; Abcam; RRID:AB_297491), anti‐CD8 (ab203035; Abcam), anti‐CD4 (ab183685; Abcam; RRID:AB_2686917), anti‐CD68 (ab125212; Abcam; RRID:AB_10975465), anti‐CD163 (ab182422; Abcam; RRID:AB_2753196), anti‐NKp46 (ab224703; Abcam), PD‐L1 (ab233482; Abcam; RRID:AB_2811045) and anti‐FoxP3 (14208S; New England Biolabs, Ipswich, MA, USA). Samples were further stained with secondary antibody (VECTASTAIN ABC Kit) at room temperature. In the next step, ABC solution (VECTASTAIN^®^ ABC‐HRP Kit, Peroxidase, Rabbit IgG, PK‐4001; Vector Laboratories) was added for 30 min, and slides were washed in 1% PBST and incubated with DAB solution (Vector DAB) for another 10 min. Sections were counterstained with haematoxylin (Sigma‐Aldrich). Results were analysed using Leica DM2500 optical microscope (Wetzlar, Germany) and presented as semi‐quantitative using imagej software (National Institutes of Health).

### 2.12 Evaluation of CXD101 monotherapy

All experiments and protocols were approved by the animal welfare body at Charles River Discovery Research Services Germany (where experiment was performed) and the local authorities, and were conducted according to all applicable international, national and local laws and guidelines. Twenty female Balb/c mice (RRID:IMSR_CRL:547) at 6–8 weeks of age (10 mice per group: control and CXD101 treated) (Charles River Laboratories, Freiburg, Germany) received unilateral subcutaneous injections of 5 × 10^5^ colon26 cells in PBS in a total injection volume of 100 µL/mouse. Upon reaching individual tumour volumes of 50–150 mm^3^, mice were assigned to treatment groups based on tumour volumes aiming at comparable group mean/median tumour volumes. Within 24 h of randomisation, mice were daily treated by oral administration (gavage) with 50 mg·kg^−1^ (dosing volume 10 mL·kg^−1^) of CXD101 using 5% DMSO/PBS as a vehicle. Body weights and tumour volume [mm^3^] were performed by calliper measurement twice weekly. Termination of individual mice was conducted at day 14 of the experiment or at > 1000 mm^3^ (unilateral), in case of tumour ulceration or body mass loss at < 70% of initial weight. From each group, four snap‐frozen tumours were collected for RNA isolation and four formalin‐fixed samples were prepared for immunohistochemical staining.

### 2.13 Evaluation of CXD101 in combination therapy

Animal welfare for this study complied with the U.S. Department of Agriculture's Animal Welfare Act (9 CFR parts 1, 2 and 3). All experimental data management and reporting procedures were in strict accordance with applicable Crown Bioscience San Diego Guidelines and Standard Operating Procedures where the study was performed.

The tumour model colon26 was implanted subcutaneously in immunocompetent BALB/c mice (Charles River Laboratories; RRID:IMSR_CRL:547). The experiment comprised six groups of six mice each, the first of which was a vehicle control group treated only with the vehicle for CXD101. The second group received CXD101 monotherapy, administered orally at a dose level of 50 mg·kg^−1^ with a 5‐day on/2‐day off schedule. Groups 3 and 4 were treated with anti‐mPD‐1 or anti‐mCTLA4 monotherapy (both Bio X Cell; RRID:SCR_004997), respectively. Both agents were administered intraperitoneally, the former twice weekly at a dose level of 5 mg·kg^−1^ and the latter on day 1 (5 mg·kg^−1^) and days 3 and 6 (2.5 mg·kg^−1^). Groups 5 and 6 received a combination of CXD101 with anti‐mPD‐1 or anti‐mCTLA4, respectively. Body weights and tumour volume [mm^3^] by calliper measurement were performed twice weekly. The treatment phase was followed by a dose‐free observation period of varying length (max. until day 38) depending on antitumour efficacy and/or condition of the mice.

The tumour model MC38 was implanted subcutaneously in immunocompetent 6–8 weeks of age female C57BL/6 mice (RRID:MGI:5658456). The experiment comprised four groups of eight mice each (unless stated otherwise), the first of which was a vehicle control group treated only with the vehicle for CXD101. The second group received CXD101 monotherapy, administered orally at a dose level of 50 mg·kg^−1^ with a 5‐day on/2‐day off schedule. Group 3 was treated with anti‐mPD‐1 monotherapy (Bio X Cell, Lebanon, NH, USA) administered intraperitoneally twice per week at a dose level of 10 mg·kg^−1^ (days 1, 6, 11, 16, 21 and 26). Group 4 received a combination of CXD101 with anti‐mPD‐1. Body weights and tumour volume [mm^3^] by calliper measurement were performed twice weekly. The treatment phase was followed by a dose‐free observation period of varying length (max. until day 38) depending on antitumour efficacy and/or condition of the mice.

### 2.14 Statistical analysis

Statistical analyses were performed using two‐tailed, unpaired Student's *t*‐test and one‐way ANOVA test with graphpad prism 8 Software (GraphPad Software; RRID:SCR_002798). Data are shown as means with SD displayed unless otherwise indicated. *P*‐values lower than 0.05 were considered significant and are labelled by asterisks (*) for *P* < 0.05, (**) for *P* < 0.01, (***) for *P* < 0.001 and (****) for *P* < 0.0001.

## 3 Results

### 3.1 CXD101 treatment causes global effects on gene expression in human cells

**Fig. 1:**
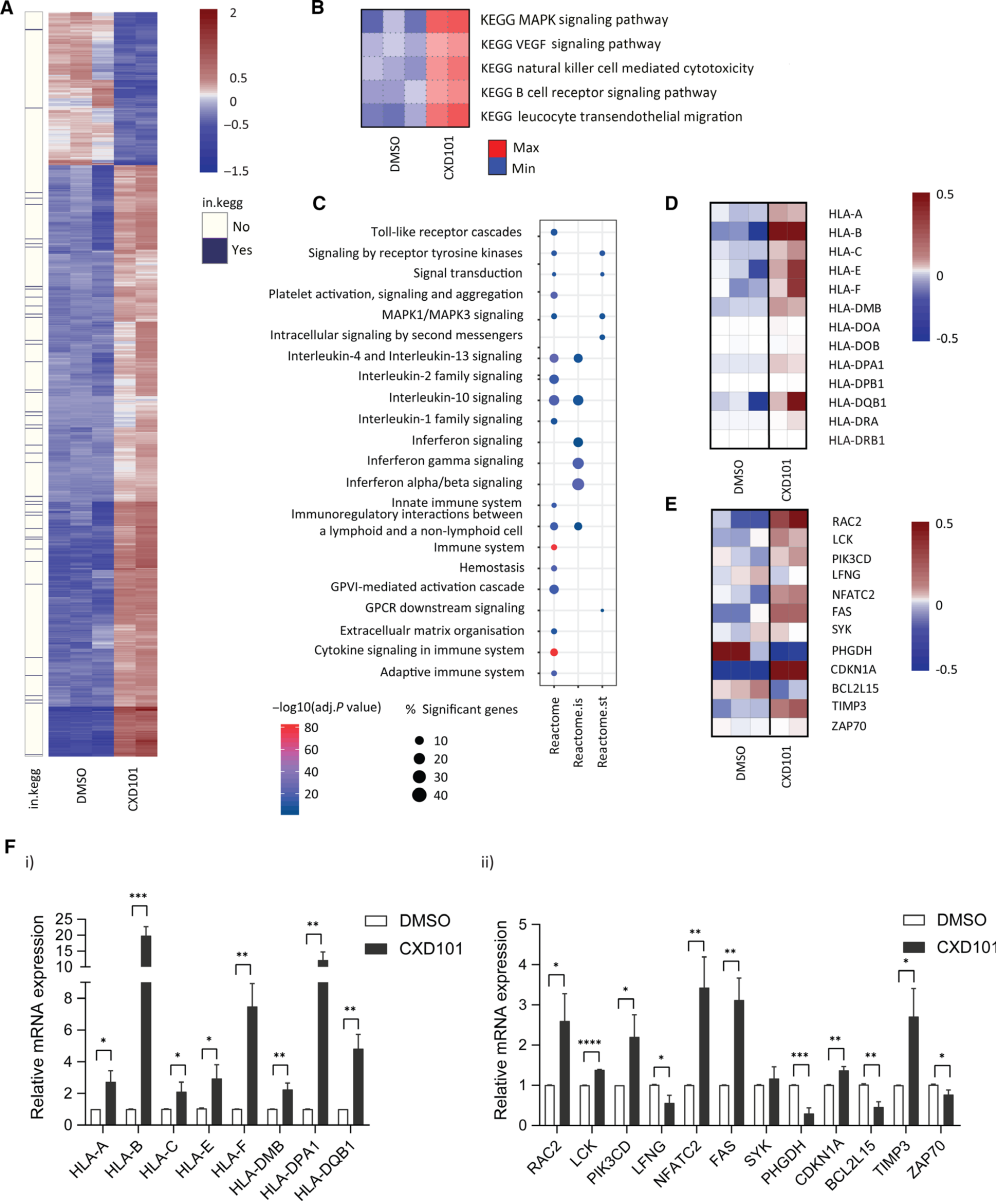
Genome‐wide analysis on CXD101‐treated SW620 cells. (A) Heat map of differential gene expression. The heat map shows 1192 significantly DEGs between CXD101‐treated cells (1 µm for 48 h; *n* = 2) and DMSO control (*n* = 3). Normalised rlog‐transformed gene expression values corresponding to significantly expressed genes (FDR < 0.01 and |log2(FC)| > 1 were mean‐centred by rows. Each row of the heat map represents transformed expression values of one DEG across all samples (blue, low expression; red, high expression). Genes associated with immune system‐related KEGG (in.kegg), pathways revealed with PGSEA (see panel B) are indicated in violet on a separate panel on the left of the heat map (see also Dataset S1). (B) Immune system‐related KEGG concepts encompassing pathways associated with significant differences in expression change upon CXD101 treatment in SW620 cell line. Heat maps show PGSEA statistic (*Z*‐score), which characterises how much the mean of the fold changes for genes in a certain pathway deviates from the mean observed in all the genes between CXD101 treatment (*n* = 2) and the control (DMSO, *n* = 3) groups. Blue indicates gene sets with decreased expression, while red corresponds to those with increased. (C) Gene enrichment analyses (pi/xgr r package) on preranked significantly DEG lists showing enriched Reactome pathways, and specifically Reactome immune system (reactome.is) and Reactome signal transduction (reactome.st) pathways. Gene ranking was performed using data sets provided and strategies implemented in xgr package. (D) Heat map showing significantly DEGs associated with Reactome immune system, referred to as the AP signature. Gene expression values were calculated as described above (see panel C). (E) Heat map of differential expression showing significantly DEGs associated with ‘Natural Killer Cell‐Mediated Cytotoxicity’ KEGG pathway. Gene expression values were transformed as described above (see panel A). (F) qRT‐PCR validation of genes identified in panels D and E (i and ii, respectively) in SW620 cells treated for 2 days with 1 µm CXD101 or DMSO control (Student's *t*‐test; *P < 0.05, error bars indicate SD); an immunoblot of SW620 cells showing the acetylation mark (H3K14) is shown in Fig. S1A(i).

We assessed the effect of CXD101 on a variety of human CRC cell lines and chose SW620 cells for further analysis because of the sensitivity to CXD101 at 72 h of treatment and coincidental increase in the level of acetylation on histone H3 lysine (K) 14 (Fig. S1A,B) [28]. In the RNA‐seq analysis, we used treatment conditions where there was minimal effect on cell viability but where an increased acetylation mark was apparent (1 µm for 48 h; Fig. S1A,B). We performed RNA‐seq on polyA‐enriched RNA prepared from CXD101‐treated SW620 cells and compared the data to the vehicle‐alone (DMSO) treatment. Sequencing reads (FASTQ format) were aligned to the reference human genome (hg19) with star aligner and analysed for differential expression using deseq2 r package [29]. The sequencing data were of high‐quality with on average 92% of the reads able to be mapped to the genome. Genes were considered to be significantly differentially expressed if the adjusted *P*‐value, calculated using the Benjamini–Hochberg method in order to minimise the false discovery rate, was less than 0.01. We further filtered significantly DEG sets to select for genes expressed at moderately high levels using a twofold change in absolute expression level. Thus, mining the RNA‐seq data for genes differentially regulated upon CXD101 treatment revealed a large number (over 1000) of candidates (Fig. 1A). The majority of them were upregulated although a significant proportion was downregulated (70% compared with 30%, respectively) (Fig. 1A). We then applied parametric gene set enrichment analyses to the normalised rlog‐transformed gene expression matrix, which disclosed enrichment of several immune‐related KEGG concepts, including natural killer (NK) cell‐mediated cytotoxicity (Fig. 1B). Inspection of DEGs for enriched KEGG concepts identified sets of genes associated with immune system (Fig. 1A; shown in violet on the separate lane on the left of the heat map).

Next, we ran a targeted gene enrichment analyses on preranked lists, which allowed us to address the relevance of the findings to immune system function and dysfunction (Fig. 1C). We found highly enriched Reactome pathway descriptors and specifically Reactome superpathways associated with the immune system and cytokine signalling in the immune system (Fig. 1C). We constructed a heat map from significant DEGs within the Reactome immune system descriptor, focussing on major histocompatibility complex (MHC) class I and class II genes, for further analysis (Fig. 1D); we refer to this as the antigen presentation (AP) signature. We did the same with significant DEGs associated with the natural killer cell‐mediated cytotoxicity KEGG pathway (Fig. 1E), from which we selected for further analysis highly expressed genes (Fig. S2A); we refer to this signature as the natural killer (NK) signature.

It was important to validate the results from the RNA‐seq and the genes assigned to the aforementioned ontologies. We therefore measured the expression of individual transcripts by qPCR in cells. Many of the MHC class I and class II genes within the AP signature were upregulated at the single gene level in SW620 cells treated with CXD101 (Fig. 1Fi). When the same set of AP signature genes was analysed in other cell lines, including the CRC HCT116 cells, breast cancer MCF7 cells and lung cancer A549 cells, CXD101 treatment caused a similar increase in gene expression (Fig. S2B–G). We also measured genes within the NK signature where many, at the single gene level, exhibited increased expression in treated cells (Fig. 1Fii). These results highlight the ability of CXD101 to regulate immune‐relevant gene expression.

### 3.2 CXD101 upregulates genes involved with immune recognition

**Fig. 2:**
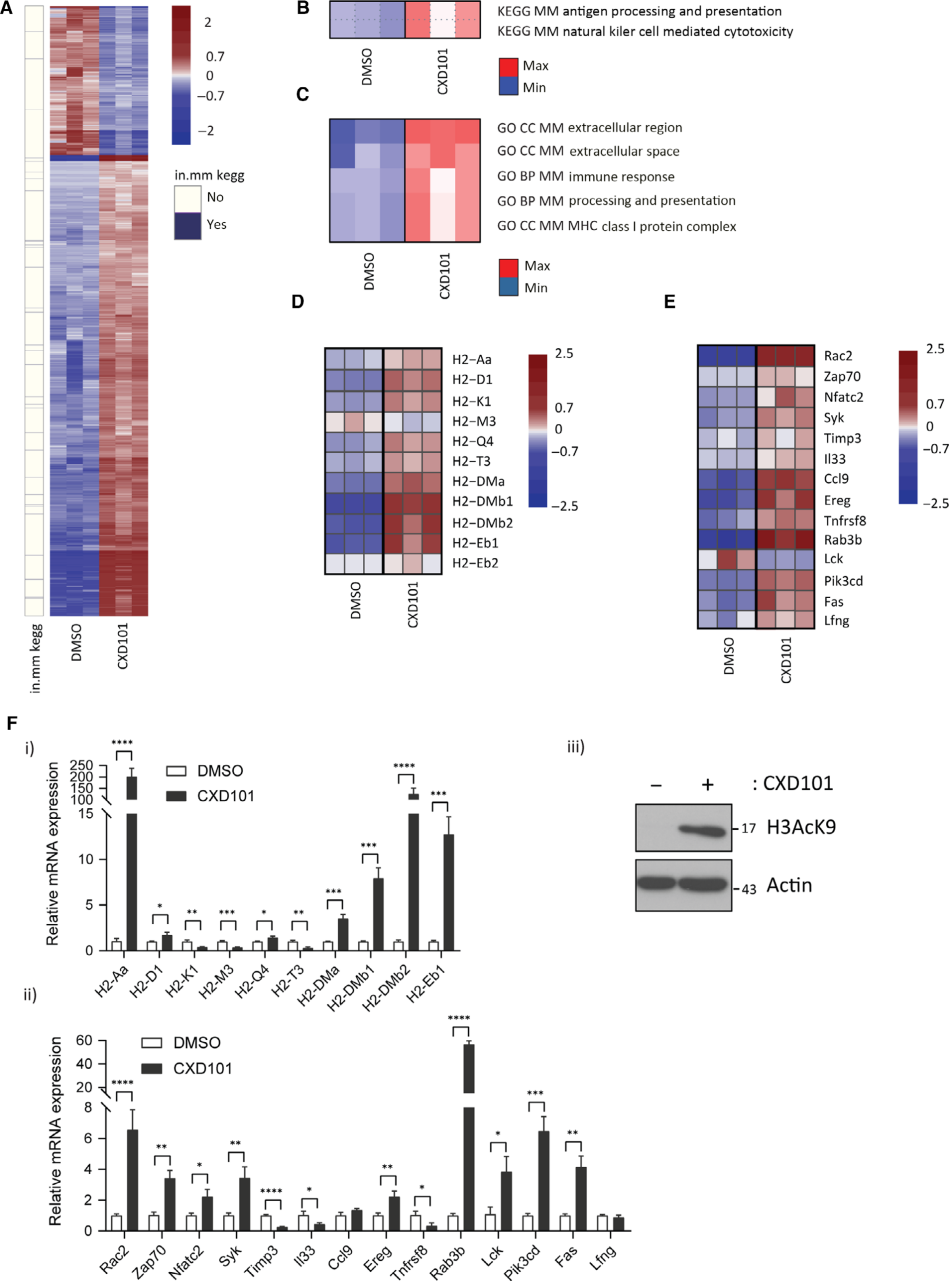
Genome‐wide analysis on CXD101‐treated colon26 cells. (A) Heat map of differential gene expression observed in colon26 cells. The heat map shows 2514 significantly DEGs between CXD101‐treated (2.7 µm for 72 h) and control (DMSO) colon26 cells. Normalised rlog‐transformed gene expression values corresponding to significantly expressed genes (FDR < 0.01 and |log2(FC)| > 1 were mean‐centred by rows. Each row of the heat map represents transformed expression values of one DEG across all samples (blue, low expression; red, high expression). Genes associated with immune system‐related KEGG pathways (see panel (B)) are indicated in violet on a separate panel (in.mm kegg) on the left of the heat map (see also Dataset S2); *n* = 3. (B) Significantly over‐represented KEGG concepts encompassing pathways associated with significant differences in expression change upon CXD101 treatment in colon26 cells. Heat maps show PGSEA statistic (*Z*‐score), which characterises how much the mean of the fold changes for genes in a certain pathway deviates from the mean observed in all the genes between CXD101 treatment and the control (DMSO) groups. Blue indicates gene sets with decreased expression, while red corresponds to those with increased; *n* = 3. (C) Significantly over‐represented GO terms encompassing biological processes or cellular components associated with significant differences in expression change upon CXD101 treatment in colon26 cells. Heat maps show PGSEA statistic (*Z*‐score), which characterises how much the mean of the fold changes for genes in a certain pathway deviates from the mean observed in all the genes between CXD101 treatment and the control (DMSO) groups. Blue indicates gene sets with decreased expression, while red corresponds to those with increased; *n* = 3. (D) Gene expression heat map showing significantly DEGs associated with ‘Antigen processing and presentation’ KEGG pathway, referred to as AP signature. Gene expression values were transformed using approach outlined above (see panel A). (E) Heat map of differential expression showing significantly DEGs associated with ‘Natural Killer Cell‐Mediated Cytotoxicity’ KEGG pathway, referred to as NK signature. Gene expression values were transformed using approach outlined above (see panel A). (F) qRT‐PCR of genes identified in panels D and E (i and ii, respectively) in colon26 cells treated for 3 days with 2.7 µm CXD101 or DMSO control (Student's *t*‐test; *P < 0.05, error bars indicate SD); (iii) an immunoblot of colon26 cells is included to demonstrate input protein levels for H3acK9; actin included as a loading control; *n* = 3.

The KEGG and Reactome analysis of DEGs in the human cell line identified gene signatures associated with antigen presentation and natural killer cells. We reasoned therefore that CXD101 *in situ* may have a wider impact perhaps on immune response to the tumour, in addition to a direct antiproliferative effect on tumour cells. We investigated this idea using the murine syngeneic colon cancer colon26 model, regarded to be MSS genomic status [30] where, initially, we studied genome‐wide expression changes in colon26 cells growing *in vitro* upon treatment with CXD101. We performed RNA‐seq on polyA‐enriched mRNA under treatment conditions where there was a CXD101‐dependent increase in histone acetylation (Fig. 2Fiii and Fig. S1C,D). Subsequently, the RNA‐seq data were aligned to the reference *Mus* *musculus* genome (mm10) with STAR aligner and analysed for differential expression. Over 90% of the reads could be mapped to the murine genome.

We constructed heat maps from the CXD101 and vehicle (DMSO)‐treated colon26 cell data, which indicated that in mouse (as in human) cells a large proportion of genes were upregulated upon CXD101 treatment (1891 upregulated and 611 downregulated; Fig. 2A). Genes found to be associated with ‘Antigen Processing and Presentation’ and ‘Natural Killer Cell‐Mediated Cytotoxicity’ KEGG pathways (Fig. 2B; referred to as AP and NK, respectively) as a result of PGSEA were attributed to ‘in.mm_kegg’ category and portrayed alongside the heat map. We could further support this statement with PGSEA‐derived GO term enrichment results, which similarly exhibited MHC‐associated gene set enrichment (Fig. 2C, and for comparison a general GO analysis, Fig. S7C). In a side‐by‐side comparison of the AP and NK KEGG pathways, the majority of genes within these categories were upregulated upon CXD101 treatment (Fig. 2D,E). For the AP category, we focussed on class I and class II genes within the murine MHC H2 gene locus, which were induced (Fig. 2D). Similarly, for the NK pathway the expression levels of many genes within the KEGG pathway were upregulated in CXD101‐treated cells (Fig. 2E). We further evaluated the expression of highly expressed genes at the single gene level by qPCR (Fig. S3A). For the AP pathway, MHC H2 class I and class II genes were induced (Fig. 2Fi). For the NK pathway, many genes were upregulated in treated cells (Fig. 2Fii).

### 3.3 Genome‐wide effects of CXD101 during tumorigenesis

To assess gene expression in the TME, we evaluated the effect of CXD101 in the syngeneic colon26 carcinoma model in tumours grown subcutaneously in Balb/c mice, with CXD101 given orally for two consecutive 5‐day periods. RNA‐seq was performed on polyA‐enriched RNA purified from the tumours. Formalin‐fixed paraffin‐embedded samples were prepared in parallel to assess by immunohistochemistry (IHC) any change in the cellular content of the TME.

**Fig. 3:**
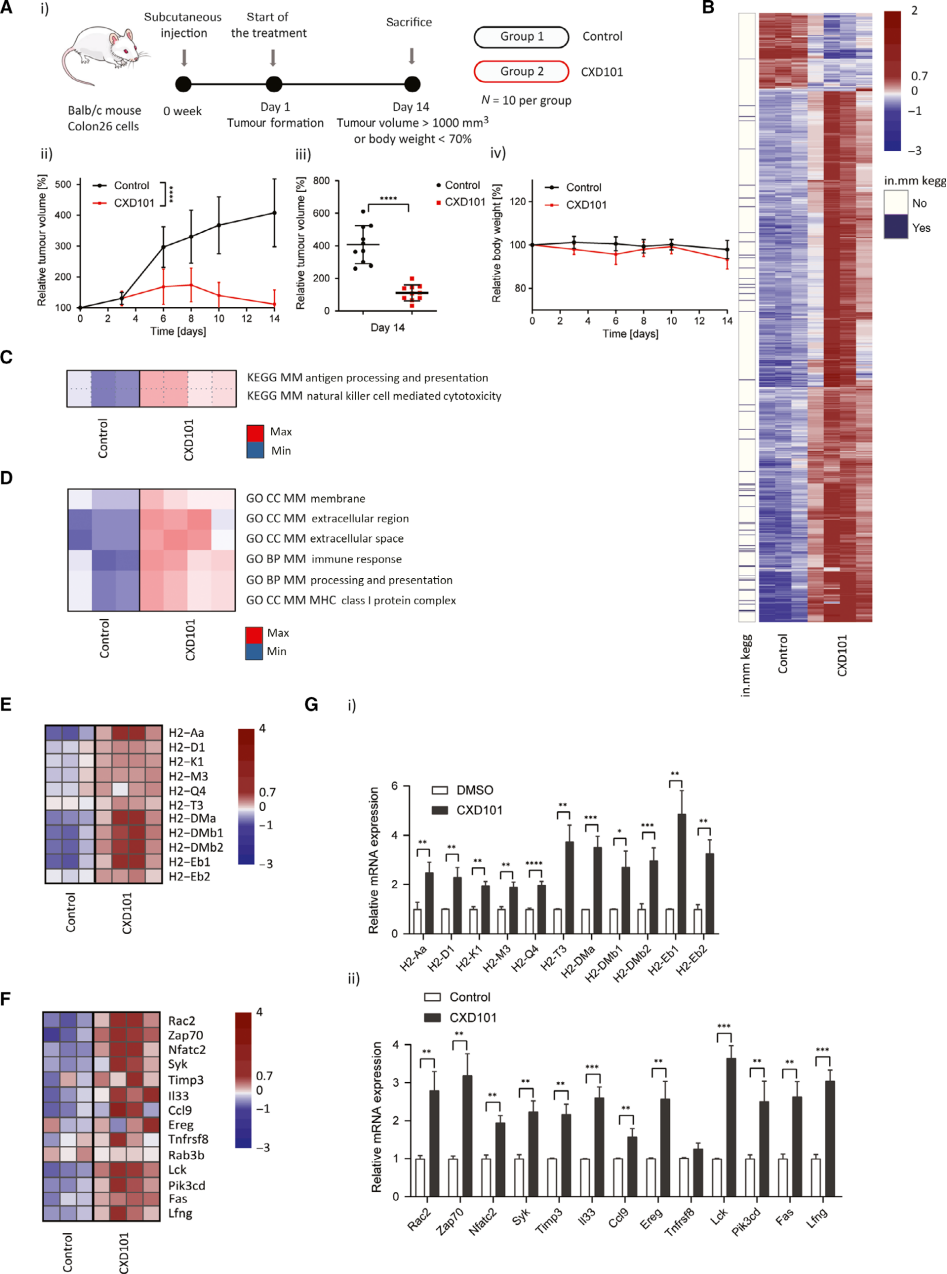
Genome‐wide analysis on CXD101‐treated colon26 tumours. (A) Schematic representation of the experiment with CXD101 in colon26 tumours (i). Balb/c mice were treated with orally administrated CXD101 at 50 mg·kg^−1^ for 14 days with respect to vehicle‐only control; *n* = 10 per group; (ii) relative tumour growth volume in CXD101‐treated and nontreated Balb/c mice presented as a mean value (Student's *t*‐test of the values at day 14; *P < 0.05); (iii) scatter plots of relative tumour volume of individual mouse at day 14 (*t*‐test; *P < 0.05); (iv) relative body weight representation of CXD101‐treated and nontreated Balb/c mice presented as a mean value. (B) Heat map of differential expression. The heat map shows 1036 significantly DEGs between CXD101‐treated (*n* = 4) and control (*n* = 3) colon26 syngeneic tumour samples. Normalised rlog‐transformed gene expression values corresponding to significantly expressed genes (FDR < 0.01 and |log2(FC)| > 1 were mean‐centred by rows). Each row of the heat map represents transformed expression values of one DEG across all samples (blue, low expression; red, high expression). Genes associated with immune system‐related KEGG pathways (see panel (C)) are indicated in violet on a separate panel (in.mm kegg) on the left of the heat map (see also Dataset S3). (C) Significantly over‐represented KEGG concepts encompassing pathways associated with significant differences in expression change upon CXD101 treatment of the tumours. Heat maps show PGSEA statistic (*Z*‐score), which characterises how much the mean of the fold changes for genes in a certain pathway deviates from the mean observed in all the genes between CXD101 treatment (*n* = 4) and the control (DMSO, *n* = 3) groups. Blue indicates gene sets with decreased expression, while red corresponds to those which were increased. (D) Significantly over‐represented GO terms encompassing biological processes or cellular components associated with significant differences in expression change upon CXD101 treatment of the tumours. Heat maps show PGSEA statistic (*Z*‐score), which characterises how much the mean of the fold changes for genes in a certain pathway deviates from the mean observed in all the genes between CXD101 treatment (*n* = 4) and the control (DMSO, *n* = 3) groups. Blue indicates gene sets with decreased expression, while red corresponds to those with increased. (E) Gene expression heat map showing significantly DEGs associated with ‘Antigen processing and presentation’ KEGG pathway, the AP signature. Gene expression values were transformed using approach outlined above (see panel B). (F) Heat map of differential expression showing significantly DEGs associated with ‘Natural Killer Cell‐Mediated Cytotoxicity’ KEGG pathway, the NK signature. Gene expression values were transformed using approach outlined above (see panel B). (G) qRT‐PCR validation of genes identified in panels E and F (i and ii, respectively) in colon26 syngeneic tumour RNA treated for 14 days with 50 mg·kg^−1^ CXD101 or DMSO control; *n* = 3 (Student's *t*‐test; *P < 0.05, error bars indicate SD).

CXD101 treatment caused a significant inhibition of tumour growth with minimal effect on body weight (Fig. 3A). The tumour RNA‐seq data were aligned to the reference *M*. *musculus* genome (mm10) with STAR aligner where over 90% of the reads mapped to the mouse genome. We analysed DEGs (|log2 FC| >1 and FDR <1%) that were upregulated and downregulated and created a heat map of the expression changes where many genes were upregulated with a smaller group downregulated upon CXD101 treatment (Fig. 3B). Similarly, PGSEA revealed a proportion of the DEGs in the tumour RNA‐seq data associated with KEGG pathways with immune‐related functions (Fig. 3B). The same two KEGG pathways namely ‘Antigen Processing and Presentation’ (AP) and ‘Natural Killer Cell‐Mediated Cytotoxicity’ (NK) were found to be enriched in the CXD101 tumour expression data (Fig. 3C). In the GO term, enrichment analysis performed with PGSEA, MHC‐associated genes and other immune‐related terms were enriched upon CXD101 treatment (Fig. 3D). In a side‐by‐side comparison of the AP and NK signatures, a majority of genes within each pathway were upregulated upon CXD101 treatment (Fig. 3E,F).

When highly expressed candidate genes within the KEGG AP pathway (Fig. S3B) were examined by qPCR at the single gene level in RNA harvested from the tumours, the expression of a range of genes within the MHC H2 complex locus was increased, including both MHC class I and class II genes (Fig. 3Gi). We also validated genes within the NK KEGG pathway, many of which were upregulated under CXD101 treatment (Fig. 3Gii). These results indicate that CXD101 upregulates immune‐relevant gene expression *in situ* in the colon26 syngeneic tumour model.

### 3.4 CXD101 regulates the cellular content of the tumour microenvironment

**Fig. 4:**
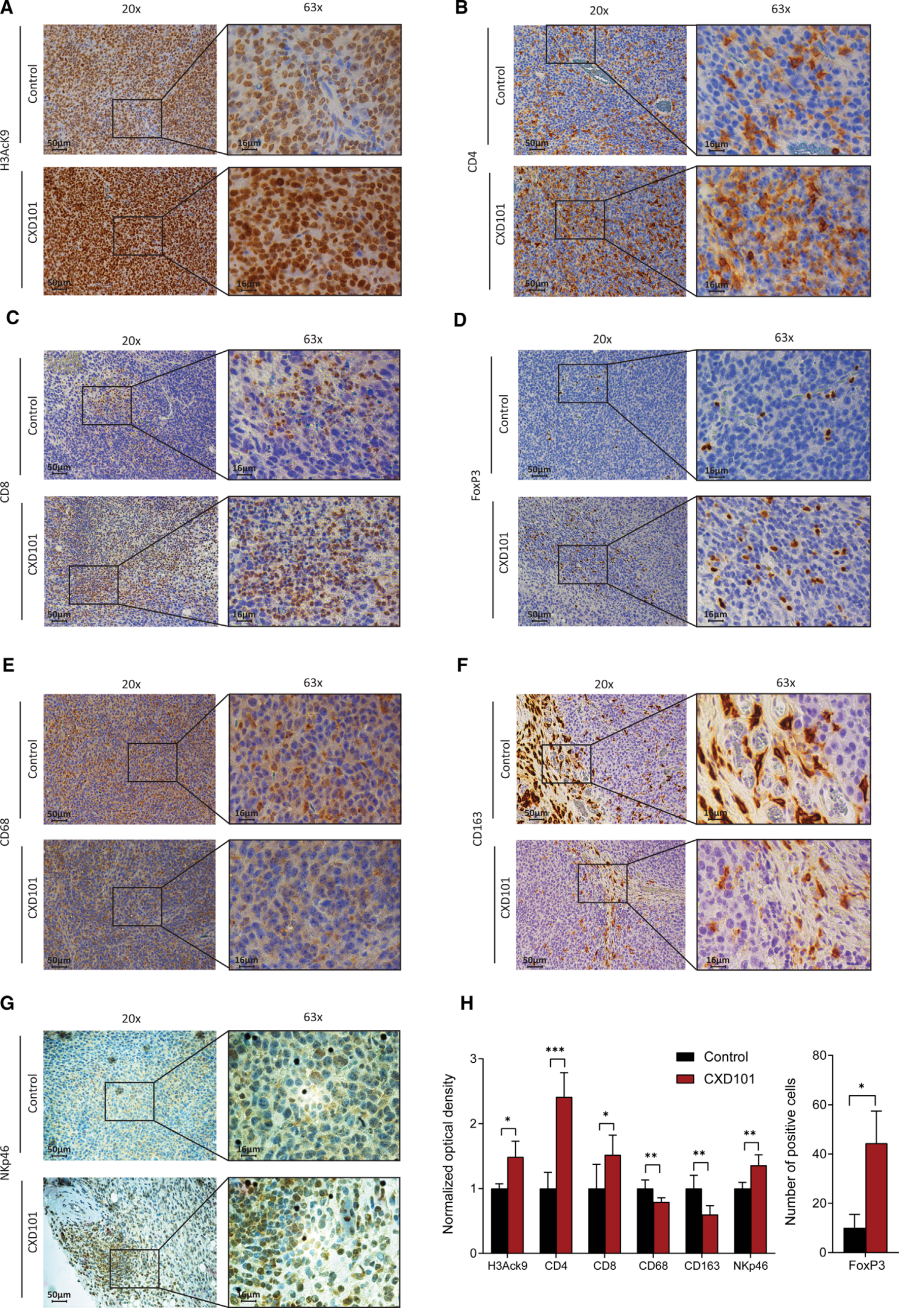
CXD101 affects the TME of colon26 tumours. (A) Representative examples of immunohistochemical staining of H3AcK9 in colon26 tumours collected from Balb/c mice at 14 days treated with 50 mg·kg^−1^ CXD101 and nontreated control (see experiment in Fig. 3A). Original magnification: 20×, scale bar, 50 μm; and 63×; scale bar, 16 μm. *n* = 4. (B) As above, but immunohistochemical staining was performed with anti‐CD4. (C) As above, but immunohistochemical staining was performed with anti‐CD8. (D) As above, but immunohistochemical staining was performed with anti‐FoxP3. (E) As above, but immunohistochemical staining was performed with anti‐CD68 gene. (F) As above, but immunohistochemical staining was performed with anti‐CD163. (G) As above, but immunohistochemical staining was performed with anti‐NKp46. (H) Results were quantified by imagej fiji software, and normalised optical density was presented as a mean ± SD. In case of Tregs (FoxP3), results were presented as an absolute number of positive cells. Statistical analysis was performed using two‐tailed, unpaired Student's *t*‐test with graphpad prism 8 software, *n* = 4.

We reasoned that if the influence of CXD101 on immune‐relevant gene expression was to be biologically important, we might expect to see evidence in the colon26 tumour model for immunological changes in the TME. We evaluated this possibility by performing IHC with markers for different lymphocyte sets and other immune‐relevant cell populations (Fig. 4). To confirm that CXD101 was active in the TME, we examined the acetylation level of histone H3 lysine (K) 9 in tumour biopsies where an increased level of nuclear H3K9 acetylation in the CXD101‐treated animals was evident (Fig. 4A,H).

We evaluated the level of helper CD4 T lymphocytes and cytotoxic CD8 T lymphocytes. Relative to the control group, there was a significant increase in the level of CD4 and in CD8 lymphocytes upon CXD101 treatment (Fig. 4B,C,H). T suppressor lymphocytes, Tregs, were assessed by measuring the level of FoxP3 positive cells [31]. Although the increase observed upon CXD101 treatment was significant, the overall level of Tregs was quite low and the immunosuppressive effect if any was likely to have been overcome by the substantial increase in infiltrating T cells (Fig. 4D,H). We also studied the level of tumour‐associated macrophages, which can take on a tumour‐promoting role [32]. By measuring the pan‐macrophage markers CD68 and CD163, a marker for the macrophages presenting immunoinhibitory features [33], we found that both populations of macrophages were reduced upon CXD101 treatment (Fig. 4E,F,H). We also measured the population of NK cells using anti‐NKp46, an established marker for NK cells [34]. A low level of NK cell staining was apparent, which was increased upon CXD101 treatment (Fig. 4G,H). Similarly, we measured the level of PD‐L1, which has been established to undergo increased expression with therapies, which sensitise tumours to ICIs [35, 36], where we found increased expression upon CXD101 treatment (Fig. S7A,B). These results establish that there are significant changes in the lymphocyte and macrophage populations in the colon26 TME upon treatment with CXD101.

### 3.5 Combined effect of CXD101 with immune checkpoint inhibitors

The ability of CXD101 to influence immune‐relevant gene expression in addition to its effect on the TME prompted us to examine the effect of CXD101 when co‐administered with ICIs, such as anti‐PD‐1 and anti‐CTLA4, which exert their anticancer activity through the immune system [37]. In this respect, it is noteworthy that colon26 tumours are poorly responsive to ICI monotherapy [38].

**Fig. 5:**
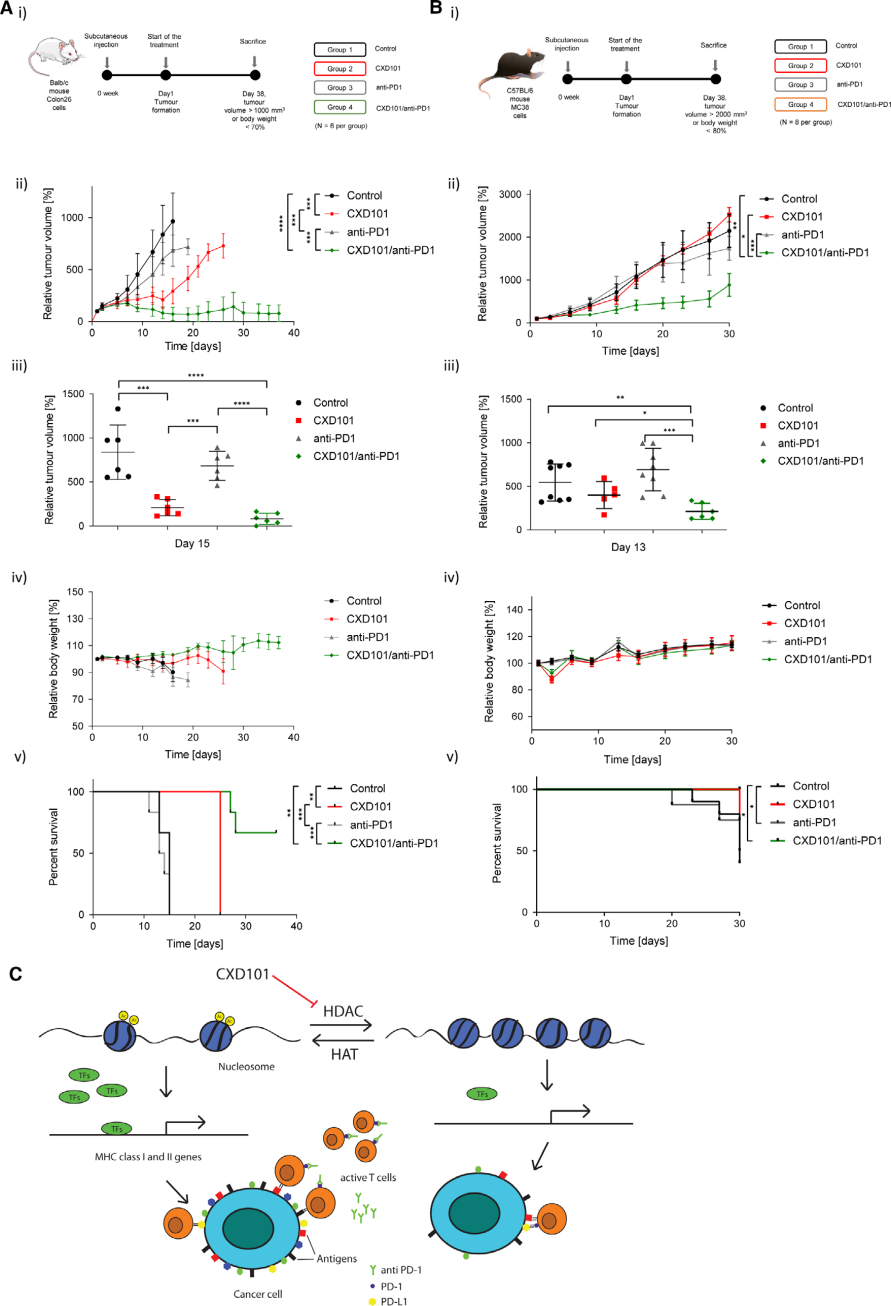
Treatment of colon26 tumours with CXD101 and anti‐PD‐1. (A) Schematic representation of the experiment with CXD101 in colon26 tumours (i). Balb/c mice were treated with orally administrated CXD101 (50 mg·kg^−1^; 5‐day on/2‐day off schedule) for 38 days or the vehicle‐only control. Additionally, group 3 was treated with anti‐mPD‐1 monotherapy administered intraperitoneally, twice weekly at a dose level of 5 mg·kg^−1^. Group 4 received a combination of CXD101 with anti‐mPD‐1; *n* = 6; (ii) scatter plots of relative tumour volume of individual mice at day 15 (Student's *t*‐test; *P < 0.05); *n* = 6; (iii) relative growth analysis of treated and nontreated tumours (Student's *t*‐test of the values at day 15; *P < 0.05); *n* = 8 (iv) relative body weight representation of treated and nontreated mice; *n* = 6; (v) survival curves of treated and nontreated mice (log‐rank [Mantel–Cox] test; *P < 0.05); *n* = 6. (B) Schematic representation of mouse experiment with CXD101 in MC38 tumours (i). C57BL/6 mice were treated with orally administrated CXD101 (50 mg·kg^−1^; 5‐day on/2‐day off schedule) for 38 days (*n* = 6) or the vehicle‐only control (*n* = 8). Group 3 mice were treated with anti‐mPD‐1 monotherapy (*n* = 8), administered intraperitoneally twice per week (10 mg·kg^−1^; days 1, 6, 11, 16, 21 and 26). Group 4 received a combination of CXD101 and anti‐mPD‐1; *n* = 6; (ii) scatter plots of relative tumour volume of individual mouse at day 13 (Student's *t*‐test; *P < 0.05); (iii) relative tumour growth analysis of treated and nontreated mice presented as a mean value (Student's *t*‐test of the values at day 13; *P < 0.05); (iv) relative body weight of treated and nontreated mice presented as a mean value; (v) survival curves of treated and nontreated mice (log‐rank (Mantel–Cox) test; *P < 0.05). (C) Model hypothesis for the effect of HDAC inhibition in regulating the immune response to tumours. It is proposed that inhibition of histone deacetylases induces expression of MHC genes and thereby increases antigen presentation, which together with the release of T cells from immune checkpoint inhibition with anti‐PD‐1 contributes to improved T‐cell engagement via MHC class I and improved tumour cell killing.

Monotherapy anti‐PD‐1 had little effect on the growth of colon26 tumours (Fig. 5Aii,iii). CXD101 treatment had an inhibitory effect on tumour growth, but the tumour began to progress at day 16 once the CXD101 dosing schedule had finished. However, when both agents were combined in a single treatment schedule, there was a marked inhibition on tumour growth, superior to each agent alone and evident after the duration of the treatment schedule had finished (Fig. 5Aii). Significantly, at a gross level the combined therapy was well‐tolerated reflecting the unaltered body weight of the animals (Fig. 5Aiv). There was an increase in overall survival in the combined compared with single treatments (Fig. 5Av) and the change in relative tumour volume was most significant in the combined treatment (Fig. 5Aiii).

The effect of combining CXD101 with anti‐CTLA4 was also investigated. Anti‐CTLA4 delayed tumour progression as a monotherapy, and the combination of CTLA4 with CXD101 was similarly significant in the enhanced antitumour activity (Fig. S4A–C). There was no reduction in body weight (Fig. S4D), and the combination significantly extended the overall survival with a clear effect on relative tumour volume (Fig. S4C,E).

We performed a similar *in vivo* tumour study using the murine syngeneic MC38 CRC model (Fig. 5B), regarded to be MSI genomic status [39]. Compared to colon26, there was less effect on MC38 tumour progression with CXD101 as a monotherapy and anti‐PD‐1 as a monotherapy behaved in a similar way. However, when administered as a combination therapy, an improved antitumour effect was observed (Fig. 5Bii,iii,v). Again, the combination did not affect body weight and had a striking effect on percentage survival and relative tumour volume (Fig. 5Bii–v). Generally, we conclude that CXD101 acts to augment the antitumour effect of ICIs like anti‐PD‐1, which exhibits a striking effect in combination with CXD101.

### 3.6 Comparison to other HDAC inhibitors

We assessed the KEGG AP and NK signatures in open‐access RNA‐seq data sets derived from other cell‐based studies with the HDAC inhibitors entinostat, vorinostat, panobinostat or chidamide [25, 26]. For the AP signature, many of the MHC class I and class II genes that were induced by CXD101 were also affected upon treating cells with the other HDAC inhibitors (Figs S5A and S6A–D). CXD101 treatment was, however, the most effective at causing increased MHC class I gene expression in a range of cancer cell lines (Fig. 1F, Fig. S2). This contrasted for example with panobinostat and chidamide, which were less effective treatments for inducing MHC class I expression (Figs S5 and S6). MHC class II genes were induced by CXD101 treatment in diverse cancer cell lines (Fig. 1F, Fig. S2), with a similar effect observed for entinostat and vorinostat (Figs S5 and S6). Panobinostat treatment had minimal effect on MHC class II expression, whereas chidamide treatment led to increased MHC class II expression, contrasting with its effect on MHC class I expression (Fig. S6).

The effect on the AP signature contrasted with the NK signature, where there were drug‐specific effects. For example, CXD101 treatment effectively induced genes within the NK signature (Figs 1F and 2F and Fig. S5). CXD101 treatment caused increased expression of RAC2, LCK, PIK3CD, FAS, NFATC2 and TIMP3, whereas entinostat treatment caused increased expression of RAC2, LCK, PIK3CD, TIMP3 and CDKN1A. Vorinostat treatment affected the expression of LCK, SYK and TIMP3, whereas panobinostat and chidamide had the least effect on the NK signature (Fig. S6). At a general level, and within the limitations of the HDAC inhibitors that we have tested and the data sources we have been able to access, we conclude that regulation of genes within the ‘antigen processing and presentation’ and ‘natural killer cell‐mediated cytotoxicity’ signatures is shared with some other HDAC inhibitors.

### 3.7 Disease association

We further explored the AP and NK signatures in a collection of public access RNA‐seq data sets derived from human malignancies and normal tissue counterparts (Xena browser [22] and cBioPortal [23, 24]). There was quite heterogeneous expression of the AP signature with normal tissues generally exhibiting low‐level expression relative to malignant tissues (Fig. S5Bi). In contrast, the NK signature was relatively poorly expressed across all samples and its expression appeared to be independent of the malignant state (Fig. S5Bi).

We examined whether there was any disease stage dependency of the AP and NK signatures, and further any influence of microsatellite status in human disease. It is noteworthy that in CRC stage IV disease, the expression of the AP signature was very low, compared with stage I through to stage III disease (Fig. S5Bii). This pattern of expression was not evident in stomach or oesophageal cancer, where stage IV disease continued to express the AP signature (Fig. S5Bii). Interestingly, when the AP signature was grouped according to microsatellite status (MSS or MSI) in CRC, there was a clear differentiation with MSS disease expressing minimal levels and MSI disease expressing high levels (Fig. S5Bii). In oesophageal cancer, the AP signature was more apparent in MSS compared with MSI disease, and in stomach cancer, there was no apparent difference between MSI and MSS status diseases (Fig. S5Bii). In contrast, the NK signature showed limited disease stage dependency apart from in stomach cancer (Fig. S5Bii). From these results, we conclude that the AP expression signature shows disease stage dependency and in some malignancies, such as CRC, relates to microsatellite status.

## 4 Discussion

Tumours can escape immune recognition through a variety of mechanisms, involving both tumour cell‐intrinsic and extracellular mechanisms [40]. Within the TME, a variety of malignant and nonmalignant cells exist [41]. Many are derived from the immune system, but also including other cells such as fibroblasts, which contribute to the stromal component [41]. Different T lymphocytes sets exist, for example cytotoxic CD8 and CD4 helper T cells [42]. Effective T‐cell immunity requires recognition of antigens presented to the immune system in the context of MHC class I and class II proteins by CD8‐positive and CD4‐positive T cells, respectively. In certain cancers, high numbers of CD8 and CD4 cells in the TME correlate with better prognosis disease [43].

The T lymphocytes most often described as tumour‐promoting are the immune‐suppressive T regulatory cells (Tregs) in part mediated through cell contact through CTLA4 (cytotoxic T lymphocyte antigen 4) [44]. CTLA4 is expressed by T cells and binds members of the B7 family expressed by antigen‐presenting cells (APCs) to inhibit T‐cell co‐stimulation during the priming and effector phases of T‐cell activation [45]. Similarly, PD‐1, a member of the extended CD28/CTLA4 family of T‐cell regulators, is expressed by activated T cells where it binds to the PD‐1 ligand expressed by tumours and APCs to inhibit T‐cell effector function, a reversible process termed T‐cell ‘exhaustion’ [46].

Many lines of evidence have established that the PD‐1 pathway and its related family members negatively regulate immune responses [47]. PD‐L1 is highly expressed in several cancers and the role of PD‐L1 in cancer immune evasion is well‐established [48]. Many tumour cells express PD‐L1, and inhibition of the interaction between PD‐1 and PD‐L1 by ICIs such as anti‐PD‐1 or PD‐L1 antibodies enhances cytotoxic T‐cell responses through engagement with MHC class I antigens [49]. The expression of PD‐L1 is correlated with reduced survival in oesophageal, pancreatic and other types of cancers [50]. In other cancers, such as CRC, clinical activity of CIs is influenced by the microsatellite status of the tumour where, generally, MSI disease is responsive to checkpoint inhibition (response rate of 25%) in contrast to MSS disease, which is generally unresponsive [51]. Consequently, there is a pressing need to develop strategies, which turn unresponsive cancers such as MSS CRC into disease that responds favourably.

The results presented here show that CXD101, an HDAC inhibitor in clinical trials [10], has widespread effects on gene expression. Most significantly, the expression of genes involved in immune recognition was increased in CRC models (both tumour cell lines grown *in vitro* and syngeneic tumour models) treated with CXD101. Specifically, two gene signatures belonging to AP and NK were increased upon CXD101 treatment, with the regulation of individual genes within each signature being confirmed at the single gene level. *In situ*, the gene expression changes reflected coincident effects on the cell populations within the TME, with CD8 and CD4 T lymphocytes undergoing a marked increase upon CXD101 treatment. Most significantly, in the colon26 and MC38 syngeneic tumour model, enhanced antitumour effects were evident upon combining CXD101 with anti‐PD‐1 ICI. At a mechanistic level, we suggest that the ability of CXD101 to induce expression of the AP signature, which includes MHC class I genes and thereby increases antigen presentation, contributes to improved cytotoxic T‐cell engagement and tumour cell killing. Hypothetically, breaking the PD‐1‐PD‐L1 checkpoint through the action of anti‐PD‐1 antibodies could release T cells, which can then engage with the increased level of MHC class I gene expression on the tumour cell via the T‐cell receptor, leading to increased levels of cytotoxicity and tumour cell killing (Fig. 5C).

Further analysis of the AP signature confirmed that the effect was shared with other HDAC inhibitors, such as entinostat and panobinostat, with the NK cell signature appearing to be more variably affected by different HDAC inhibitors. In this respect, our results are consistent with studies on other HDAC inhibitors demonstrating beneficial effects on the immune response, including effects on MHC gene expression [36, 52, 53, 54, 55, 56, 57] In malignant disease, the AP signature was absent in CRC stage IV disease, with a clear dichotomy evident between MSI status and MSS status, where MSS disease exhibited low expression of the AP signature in contrast to MSI.

## 5 Conclusions

In conclusion, our study has established a novel property of CXD101 relating to its ability to reinstate immune‐relevant gene expression in tumour cells. In turn, this activity coincides with the ability of CXD101 to influence the TME and to act in combination with agents such as anti‐PD‐1 to assist an effective antitumour response. We suggest that our results provide a compelling rational scientific basis for pursuing a clinical study combining CXD101 with ICIs like anti‐PD‐1, and in this respect note that a human clinical trial is underway [58]. We hypothesise that the underlying changes in immune‐relevant gene expression and consequent re‐engagement of the immune system will lead to new clinical utilities for HDAC inhibitor‐based therapies.

## Conflict of interest

The authors declare no conflict of interest.

## Author contributions

WB and GL made an equal contribution. NBLT conceptualised the data, curated the data, acquired data and supervised the data; WB, GL, WB2 and AS involved in formal analysis; WB, HZ, GL, AS and GA investigated the data; WB, HZ, GL, AS and GA designed methodology; SZ and DK provided resources; AS2 and WB2 involved in bioinformatic analysis; WB, HZ, GL, AS and GA validated the data; WB2 and AS2 visualised the data; WB and NBLT wrote the original draft; NBLT, GL and WB wrote, reviewed and edited the manuscript.

## Supporting information


**Fig. S1**. Effect of CXD101 on SW620 and colon26 colon cancer cells.

**Fig. S2**. Effect of CXD101 on genes within the AP and NK signature in SW620, MCF7, A549, and HCT116 cells.

**Fig. S3**. Effect of CXD101 on genes in the AP and NK signatures in colon26 cells *in vitro* and *in vivo*.

**Fig. S4**. Treatment with CXD101 and anti‐CTLA4 in colon26 tumours.

**Fig. S5**. Comparison with other HDAC inhibitors and disease spectrum.

**Fig. S6**. Comparison with other HDAC inhibitors and disease spectrum.

**Fig. S7**. Effect of CXD101 on PD‐L1 expression in colon26 syngeneic mouse model and general gene ontology analysis.

**Dataset S1.** List of up‐ and down‐regulated genes after HDAC inhibition in SW620 cells identified from the RNA‐seq analysis.

**Dataset S2.** List of up‐ and down‐regulated genes after HDAC inhibition in colon26 cells identified from the RNA‐seq analysis.

**Dataset S3.** List of up‐ and down‐regulated genes after HDAC inhibition in colon26 tumours *in vivo* identified from the RNA‐seq analysis.

Supplementary Material

## Data Availability

All data generated or analysed during this study are included in this published article (and its Supporting information files). Gene expression data have been deposited in NCBI's Gene Expression Omnibus and are accessible through GEO Series (RRID:SCR_005012) Accession Number GSE158164.

## References

[1] Roth SY , Denu JM & Allis CD (2001) Histone acetyltransferases. Annu Rev Biochem 70, 81–120. 11395403 10.1146/annurev.biochem.70.1.81

[2] Seto E & Yoshida M (2014) Erasers of histone acetylation: the histone deacetylase enzymes. Cold Spring Harb Perspect Biol 6, a018713. 24691964 10.1101/cshperspect.a018713 PMC3970420

[3] Marmorstein R & Zhou MM (2014) Writers and readers of histone acetylation: structure, mechanism, and inhibition. Cold Spring Harb Perspect Biol 6, a018762. 10.1101/cshperspect.a018762 24984779 PMC4067988

[4] Dawson MA & Kouzarides T (2012) Cancer epigenetics: from mechanism to therapy. Cell 150, 12–27. 22770212 10.1016/j.cell.2012.06.013

[5] West AC & Johnstone RW (2014) New and emerging HDAC inhibitors for cancer treatment. J Clin Invest 124, 30–39. 24382387 10.1172/JCI69738 PMC3871231

[6] Cappellacci L , Perinelli DR , Maggi F , Grifantini M & Petrelli R (2020) Recent progress in histone deacetylase inhibitors as anticancer agents. Curr Med Chem 27, 2449–2493. 30332940 10.2174/0929867325666181016163110

[7] Hogg SJ , Beavis PA , Dawson MA & Johnstone RW (2020) Targeting the epigenetic regulation of antitumour immunity. Nat Rev Drug Discov 19, 776–800. 32929243 10.1038/s41573-020-0077-5

[8] Shi Y , Dong M , Hong X , Zhang W , Feng J , Zhu J , Yu L , Ke X , Huang H , Shen Z *et al*. (2015) Results from a multicenter, open‐label, pivotal phase II study of chidamide in relapsed or refractory peripheral T‐cell lymphoma. Ann Oncol 26, 1766–1771. 26105599 10.1093/annonc/mdv237

[9] Park SY & Kim JS (2020) A short guide to histone deacetylases including recent progress on class II enzymes. Exp Mol Med 52, 204–212. 32071378 10.1038/s12276-020-0382-4 PMC7062823

[10] Eyre TA , Collins GP , Gupta A , Coupe N , Sheikh S , Whittaker J , Wang LM , Campo L , Soilleux E , Tysoe F *et al*. (2019) A phase 1 study to assess the safety, tolerability, and pharmacokinetics of CXD101 in patients with advanced cancer. Cancer 125, 99–108. 30332497 10.1002/cncr.31791

[11] Eyre TA (2018) Predictive biomarkers for disease sensitivity in lymphoma – the holy grail for HDAC inhibitors? Oncotarget 9, 37280–37281. 30647865 10.18632/oncotarget.26460 PMC6324661

[12] Ramos‐Casals M , Brahmer JR , Callahan MK , Flores‐Chavez A , Keegan N , Khamashta MA , Lambotte O , Mariette X , Prat A & Suarez‐Almazor ME (2020) Immune‐related adverse events of checkpoint inhibitors. Nat Rev Dis Primers 6, 38. 32382051 10.1038/s41572-020-0160-6 PMC9728094

[13] Dobin A , Davis CA , Schlesinger F , Drenkow J , Zaleski C , Jha S , Batut P , Chaisson M & Gingeras TR (2013) STAR: ultrafast universal RNA‐seq aligner. Bioinformatics 29, 15–21. 23104886 10.1093/bioinformatics/bts635 PMC3530905

[14] Love MI , Huber W & Anders S (2014) Moderated estimation of fold change and dispersion for RNA‐seq data with DESeq2. Genome Biol 15, 550. 10.1186/s13059-014-0550-8 25516281 PMC4302049

[15] Fang H , the ULTRA‐DD Consortium & Knight JC (2019) Pi: an R/Bioconductor package leveraging genetic evidence to prioritise drug targets at the gene and pathway level. Bioconductor. 10.18129/B9.bioc.Pi

[16] Fang H , Knezevic B , Burnham KL & Knight JC (2016) XGR software for enhanced interpretation of genomic summary data, illustrated by application to immunological traits. Genome Med 8, 129. 10.1186/s13073-016-0384-y 27964755 PMC5154134

[17] Fabregat A , Jupe S , Matthews L , Sidiropoulos K , Gillespie M , Garapati P , Haw R , Jassal B , Korninger F , May B *et al*. (2018) The reactome pathway knowledgebase. Nucleic Acids Res 46, D649–D655. 29145629 10.1093/nar/gkx1132 PMC5753187

[18] Furge KDK (2018) Parametric gene set enrichment analysis. R package version 1.58.0. Bioconductor. 10.18129/B9.bioc.PGSEA

[19] Ritchie ME , Phipson B , Wu D , Hu YF , Law CW , Shi W & Smyth GK (2015) limma powers differential expression analyses for RNA‐sequencing and microarray studies. Nucleic Acids Res 43, e47. 10.1093/nar/gkv007 25605792 PMC4402510

[20] Benjamini Y & Hochberg Y (1995) Controlling the false discovery rate – a practical and powerful approach to multiple testing. J R Stat Soc B 57, 289–300.

[21] Bares V & Ge X (2019) gskb: Gene Set data for pathway analysis in mouse. R package version 1.18.0. Bioconductor. 10.18129/B9.bioc.gskb

[22] Goldman MJ , Craft B , Hastie M , Repecka K , McDade F , Kamath A , Banerjee A , Luo YH , Rogers D , Brooks AN *et al*. (2020) Visualizing and interpreting cancer genomics data via the Xena platform. Nat Biotechnol 38, 675–678. 32444850 10.1038/s41587-020-0546-8 PMC7386072

[23] Cerami E , Gao JJ , Dogrusoz U , Gross BE , Sumer SO , Aksoy BA , Jacobsen A , Byrne CJ , Heuer ML , Larsson E *et al*. (2012) The cBio Cancer Genomics Portal: an open platform for exploring multidimensional cancer genomics data. Cancer Discov 2, 401–404. 22588877 10.1158/2159-8290.CD-12-0095 PMC3956037

[24] Gao JJ , Aksoy BA , Dogrusoz U , Dresdner G , Gross B , Sumer SO , Sun YC , Jacobsen A , Sinha R , Larsson E *et al*. (2013) Integrative analysis of complex cancer genomics and clinical profiles using the cBioPortal. Sci Signal 6, pl1. 10.1126/scisignal.2004088 23550210 PMC4160307

[25] Subramanian A , Narayan R , Corsello SM , Peck DD , Natoli TE , Lu XD , Gould J , Davis JF , Tubelli AA , Asiedu JK *et al*. (2017) A next generation connectivity map: L1000 platform and the first 1,000,000 profiles. Cell 171, 1437–1452. 29195078 10.1016/j.cell.2017.10.049 PMC5990023

[26] Kubo M , Kanaya N , Petrossian K , Ye J , Warden C , Liu Z , Nishimura R , Osako T , Okido M , Shimada K *et al*. (2013) Inhibition of the proliferation of acquired aromatase inhibitor‐resistant breast cancer cells by histone deacetylase inhibitor LBH589 (panobinostat). Breast Cancer Res Treat 137, 93–107. 23160924 10.1007/s10549-012-2332-x PMC3637924

[27] Ghandi M , Huang FW , Jane‐Valbuena J , Kryukov GV , Lo CC , McDonald ER , Barretina J , Gelfand ET , Bielski CM , Li H *et al*. (2019) Next‐generation characterization of the Cancer Cell Line Encyclopedia. Nature 569, 503–508. 31068700 10.1038/s41586-019-1186-3 PMC6697103

[28] Drogaris P , Villeneuve V , Pomies C , Lee EH , Bourdeau V , Bonneil E , Ferbeyre G , Verreault A & Thibault P (2012) Histone deacetylase inhibitors globally enhance H3/H4 tail acetylation without affecting H3 lysine 56 acetylation. Sci Rep 2, 220. 10.1038/srep00220 22355734 PMC3256565

[29] Love MI , Huber W & Anders S (2014) Moderated estimation of fold change and dispersion for RNA‐seq data with DESeq2. Genome Biology 15, 550. 10.1186/s13059-014-0550-8 25516281 PMC4302049

[30] Castle JC , Loewer M , Boegel S , de Graaf J , Bender C , Tadmor AD , Boisguerin V , Bukur T , Sorn P , Paret C *et al*. (2014) Immunomic, genomic and transcriptomic characterization of CT26 colorectal carcinoma. BMC Genom 15. 10.1186/1471-2164-15-190 PMC4007559 24621249

[31] Hori S , Nomura T & Sakaguchi S (2017) Pillars article: control of regulatory T cell development by the transcription factor Foxp3. Science 2003. 299: 1057–1061. J Immunol 198, 981–985. 28115586

[32] Cassetta L & Pollard JW (2018) Targeting macrophages: therapeutic approaches in cancer. Nat Rev Drug Discov 17, 887–904. 30361552 10.1038/nrd.2018.169

[33] Bertani FR , Mozetic P , Fioramonti M , Iuliani M , Ribelli G , Pantano F , Santini D , Tonini G , Trombetta M , Businaro L *et al*. (2017) Classification of M1/M2‐polarized human macrophages by label‐free hyperspectral reflectance confocal microscopy and multivariate analysis. Sci Rep 7, 8965. 28827726 10.1038/s41598-017-08121-8 PMC5566322

[34] Jelencic V , Sestan M , Kavazovic I , Lenartic M , Marinovic S , Holmes TD , Prchal‐Murphy M , Lisnic B , Sexl V , Bryceson YT *et al*. (2018) NK cell receptor NKG2D sets activation threshold for the NCR1 receptor early in NK cell development. Nat Immunol 19, 1083–1092. 30224819 10.1038/s41590-018-0209-9 PMC6166863

[35] Iwasa M , Harada T , Oda A , Bat‐Erdene A , Teramachi J , Tenshin H , Ashtar M , Oura M , Sogabe K , Udaka K *et al*. (2019) PD‐L1 upregulation in myeloma cells by panobinostat in combination with interferon‐gamma. Oncotarget 10, 1903–1917. 30956773 10.18632/oncotarget.26726 PMC6443002

[36] Woods DM , Sodre AL , Villagra A , Sarnaik A , Sotomayor EM & Weber J (2015) HDAC inhibition upregulates PD‐1 ligands in melanoma and augments immunotherapy with PD‐1 blockade. Cancer Immunol Res 3, 1375–1385. 26297712 10.1158/2326-6066.CIR-15-0077-T PMC4674300

[37] Havel JJ , Chowell D & Chan TA (2019) The evolving landscape of biomarkers for checkpoint inhibitor immunotherapy. Nat Rev Cancer 19, 133–150. 30755690 10.1038/s41568-019-0116-x PMC6705396

[38] Wilkinson RW & Leishman AJ (2018) Further advances in cancer immunotherapy: going beyond checkpoint blockade. Front Immunol 9, 1082. 10.3389/fimmu.2018.01082 29910800 PMC5992967

[39] Efremova M , Rieder D , Klepsch V , Charoentong P , Finotello F , Hackl H , Hermann‐Kleiter N , Lower M , Baier G , Krogsdam A *et al*. (2018) Targeting immune checkpoints potentiates immunoediting and changes the dynamics of tumor evolution. Nat Commun 9, 32. 10.1038/s41467-017-02424-0 29296022 PMC5750210

[40] Varade J , Magadan S & Gonzalez‐Fernandez A (2021) Human immunology and immunotherapy: main achievements and challenges. Cell Mol Immunol 18, 805–828. 32879472 10.1038/s41423-020-00530-6 PMC7463107

[41] Jin MZ & Jin WL (2020) The updated landscape of tumor microenvironment and drug repurposing. Signal Transduct Tar 5, 166. 10.1038/s41392-020-00280-x PMC7447642 32843638

[42] Tay RE , Richardson EK & Toh HC (2020) Revisiting the role of CD4(+)T cells in cancer immunotherapy‐new insights into old paradigms. Cancer Gene Ther 28, 5–17. 10.1038/s41417-020-0183-x 32457487 PMC7886651

[43] Barnes TA & Amir E (2017) HYPE or HOPE: the prognostic value of infiltrating immune cells in cancer. Br J Cancer 117, 451–460. 28704840 10.1038/bjc.2017.220 PMC5558691

[44] Walker LSK (2013) Treg and CTLA‐4: two intertwining pathways to immune tolerance. J Autoimmun 45, 49–57. 23849743 10.1016/j.jaut.2013.06.006 PMC3989116

[45] Gaudino SJ & Kumar P (2019) Cross‐talk between antigen presenting cells and T cells impacts intestinal homeostasis, bacterial infections, and tumorigenesis. Front Immunol 10, 360. 10.3389/fimmu.2019.00360 30894857 PMC6414782

[46] Wherry EJ (2011) T cell exhaustion. Nat Immunol 12, 492–499. 21739672 10.1038/ni.2035

[47] Jenkins RW , Barbie DA & Flaherty KT (2018) Mechanisms of resistance to immune checkpoint inhibitors. Br J Cancer 118, 9–16. 29319049 10.1038/bjc.2017.434 PMC5765236

[48] Vinay DS , Ryan EP , Pawelec G , Talib WH , Stagg J , Elkord E , Lichtor T , Decker WK , Whelan RL , Kumara HMCS *et al*. (2015) Immune evasion in cancer: Mechanistic basis and therapeutic strategies. Semin Cancer Biol 35, S185–S198. 25818339 10.1016/j.semcancer.2015.03.004

[49] He X & Xu CQ (2020) Immune checkpoint signaling and cancer immunotherapy. Cell Res 30, 660–669. 32467592 10.1038/s41422-020-0343-4 PMC7395714

[50] Alsaab HO , Sau S , Alzhrani R , Tatiparti K , Bhise K , Kashaw SK & Iyer AK (2017) PD‐1 and PD‐L1 checkpoint signaling inhibition for cancer immunotherapy: mechanism, combinations, and clinical outcome. Front Pharmacol 8, 561. 10.3389/fphar.2017.00561 28878676 PMC5572324

[51] Oliveira AF , Bretes L & Furtado I (2019) Review of PD‐1/PD‐L1 inhibitors in metastatic dMMR/MSI‐H colorectal cancer. Front Oncol 9, 396. 10.3389/fonc.2019.00396 31139574 PMC6527887

[52] Burke B , Eden C , Perez C , Belshoff A , Hart S , Plaza‐Rojas L , Delos Reyes M , Prajapati K , Voelkel‐Johnson C , Henry E *et al*. (2020) Inhibition of histone deacetylase (HDAC) enhances checkpoint blockade efficacy by rendering bladder cancer cells visible for T cell‐mediated destruction. Front Oncol 10, 699. 10.3389/fonc.2020.00699 32500025 PMC7243798

[53] Orillion A , Hashimoto A , Damayanti N , Shen L , Adelaiye‐Ogala R , Arisa S , Chintala S , Ordentlich P , Kao CA , Elzey B *et al*. (2017) Entinostat neutralizes myeloid‐derived suppressor cells and enhances the antitumor effect of PD‐1 inhibition in murine models of lung and renal cell carcinoma. Clin Cancer Res 23, 5187–5201. 28698201 10.1158/1078-0432.CCR-17-0741 PMC5723438

[54] Smith HJ , McCaw TR , Londono AI , Katre AA , Meza‐Perez S , Yang ES , Forero A , Buchsbaum DJ , Randall TD , Straughn JM *et al*. (2018) The antitumor effects of entinostat in ovarian cancer require adaptive immunity. Cancer 124, 4657–4666. 30423192 10.1002/cncr.31761 PMC6294677

[55] Wang XG , Waschke BC , Woolaver RA , Chen ZG , Zhang G , Piscopio AD , Liu XD & Wang JH (2019) Histone deacetylase inhibition sensitizes PD1 blockade‐resistant B‐cell lymphomas. Cancer Immunol Res 7, 1318–1331. 31235619 10.1158/2326-6066.CIR-18-0875 PMC6679731

[56] West AC , Smyth MJ & Johnstone RW (2014) The anticancer effects of HDAC inhibitors require the immune system. Oncoimmunology 3, e27414. 10.4161/onci.27414 24701376 PMC3962507

[57] Zheng H , Zhao W , Yan C , Watson CC , Massengill M , Xie M , Massengill C , Noyes DR , Martinez GV , Afzal R *et al*. (2016) HDAC inhibitors enhance T‐cell chemokine expression and augment response to PD‐1 immunotherapy in lung adenocarcinoma. Clin Cancer Res 22, 4119–4132. 26964571 10.1158/1078-0432.CCR-15-2584 PMC4987196

[58] https://clinicaltrials.gov/ct2/show/study/NCT03993626

