# Supplementary figures and images for: Immune modulation underpins the anti‐cancer activity of HDAC inhibitors
Source: Mol Oncol. 2021 May 1;15(12):3280–98. doi: 10.1002/1878-0261.12953 (PMC8637571; doi:10.1002/1878-0261.12953)

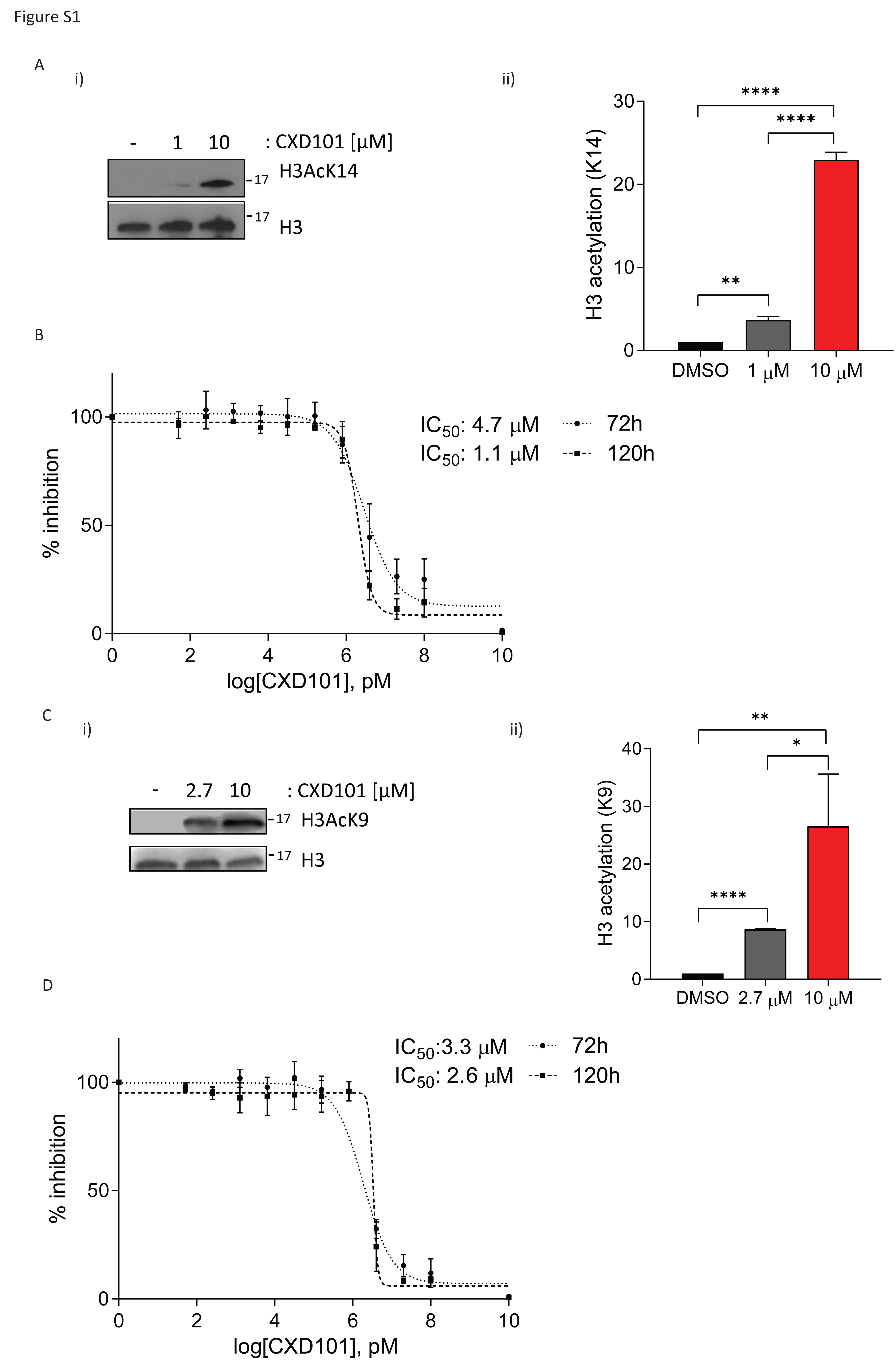

Supplement: Supplementary file 1 — Fig. S1. Effect of CXD101 on SW620 and colon26 colon cancer cells. [file MOL2-15-3280-s001.tif]

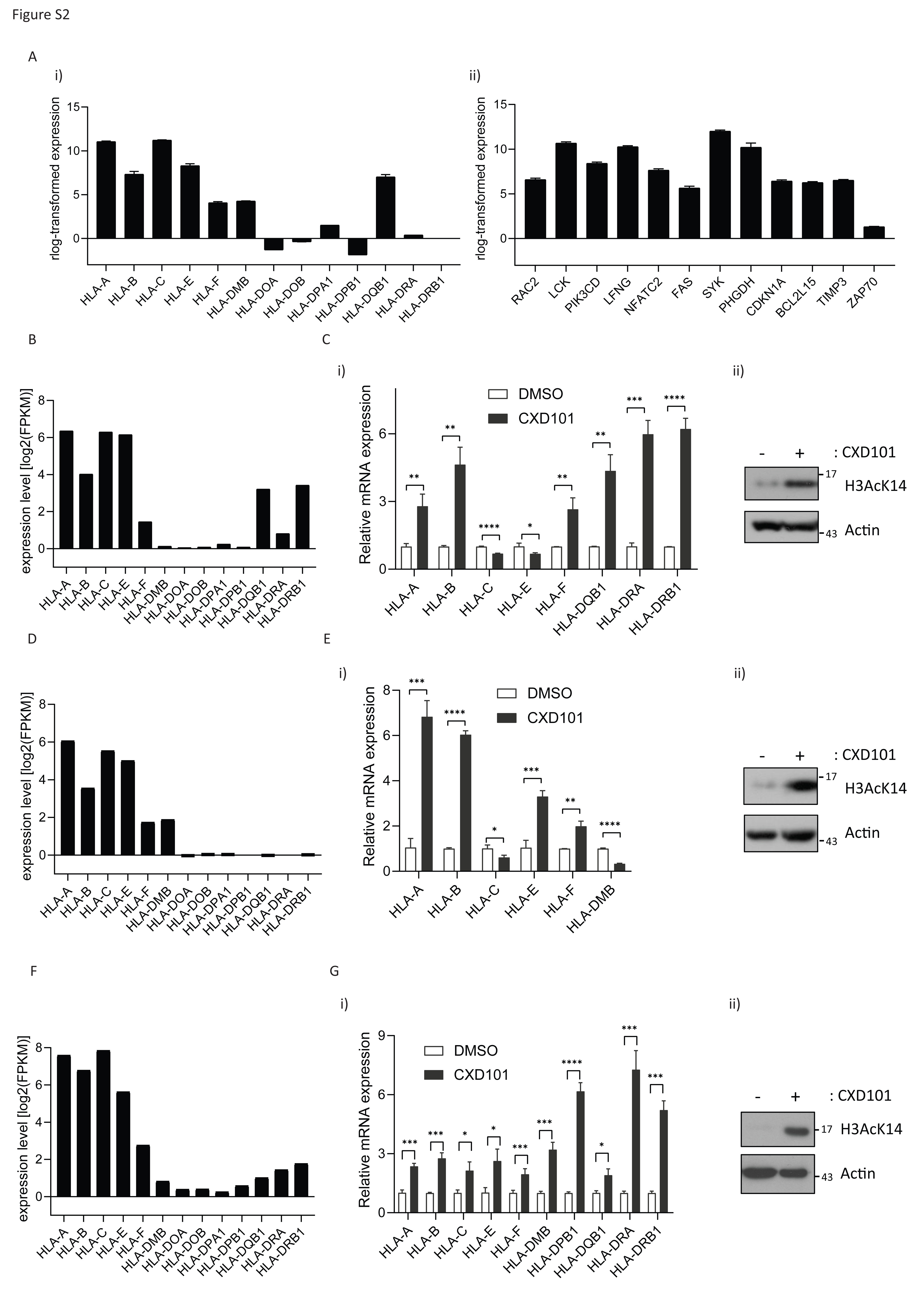

Supplement: Supplementary file 2 — Fig. S2. Effect of CXD101 on genes within the AP and NK signature in SW620, MCF7, A549, and HCT116 cells. [file MOL2-15-3280-s005.tif]

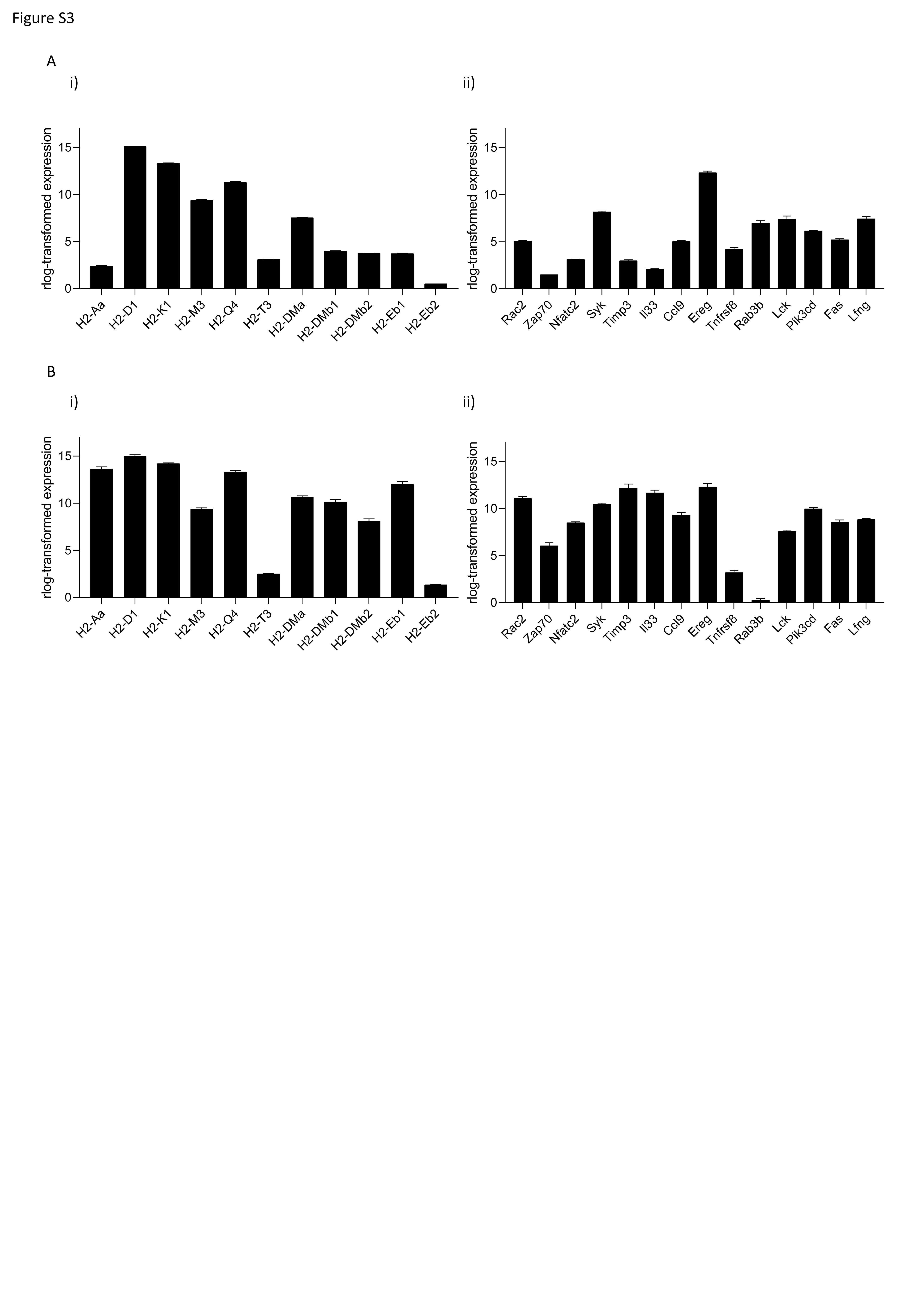

Supplement: Supplementary file 3 — Fig. S3. Effect of CXD101 on genes in the AP and NK signatures in colon26 cells in vitro and in vivo. [file MOL2-15-3280-s011.tif]

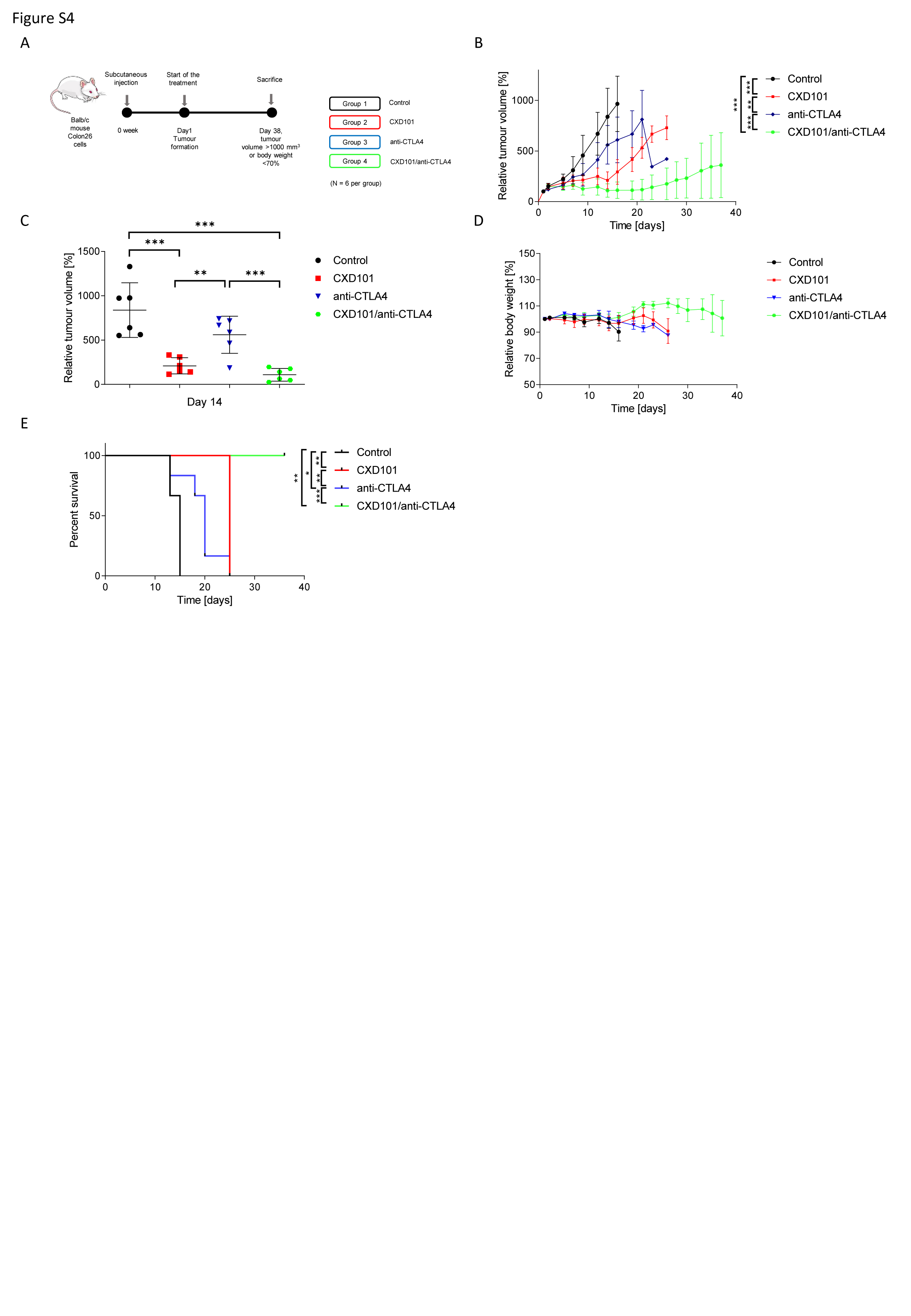

Supplement: Supplementary file 4 — Fig. S4. Treatment with CXD101 and anti‐CTLA4 in colon26 tumours. [file MOL2-15-3280-s009.tif]

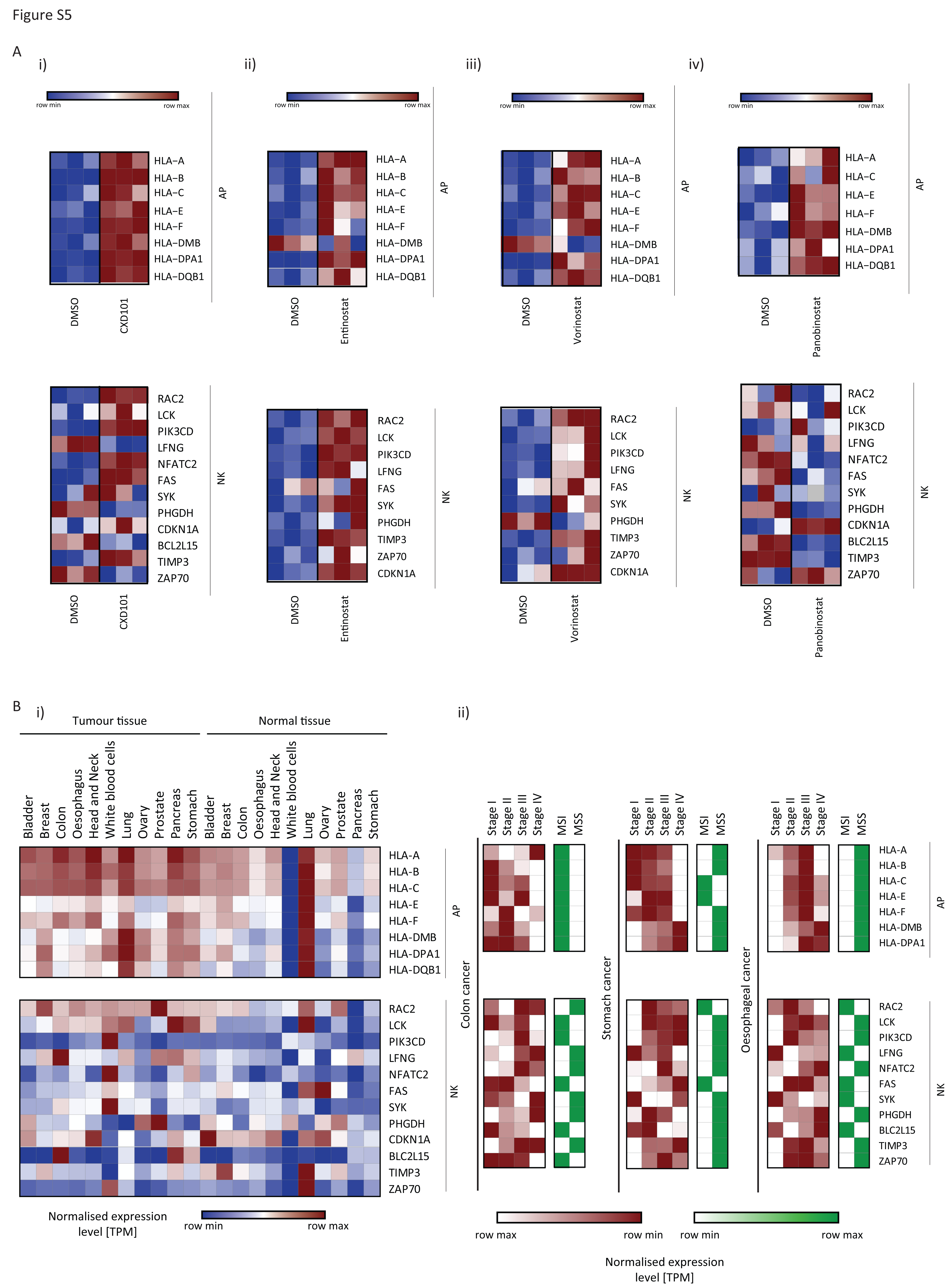

Supplement: Supplementary file 5 — Fig. S5. Comparison with other HDAC inhibitors and disease spectrum. [file MOL2-15-3280-s007.tif]

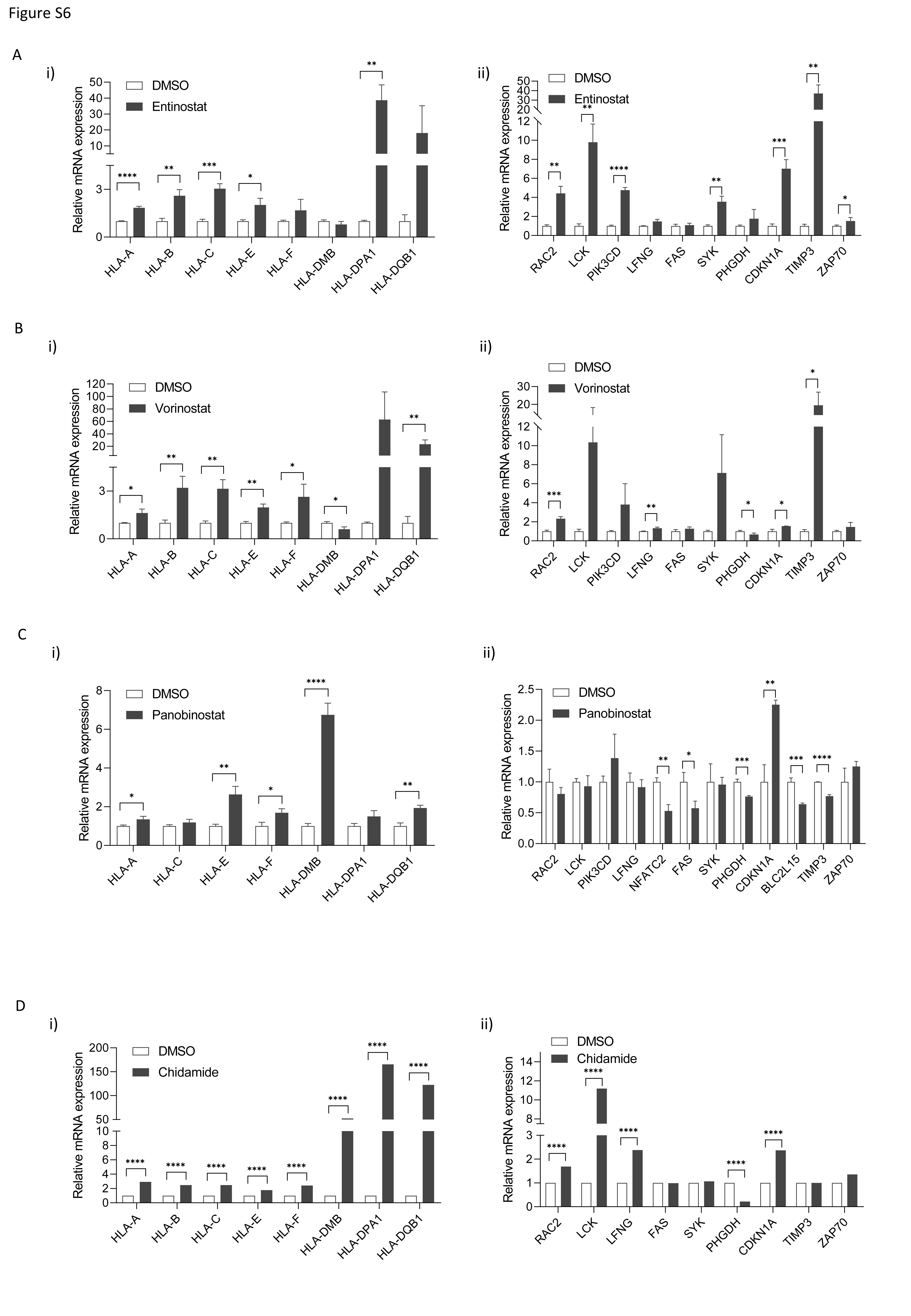

Supplement: Supplementary file 6 — Fig. S6. Comparison with other HDAC inhibitors and disease spectrum. [file MOL2-15-3280-s002.tif]

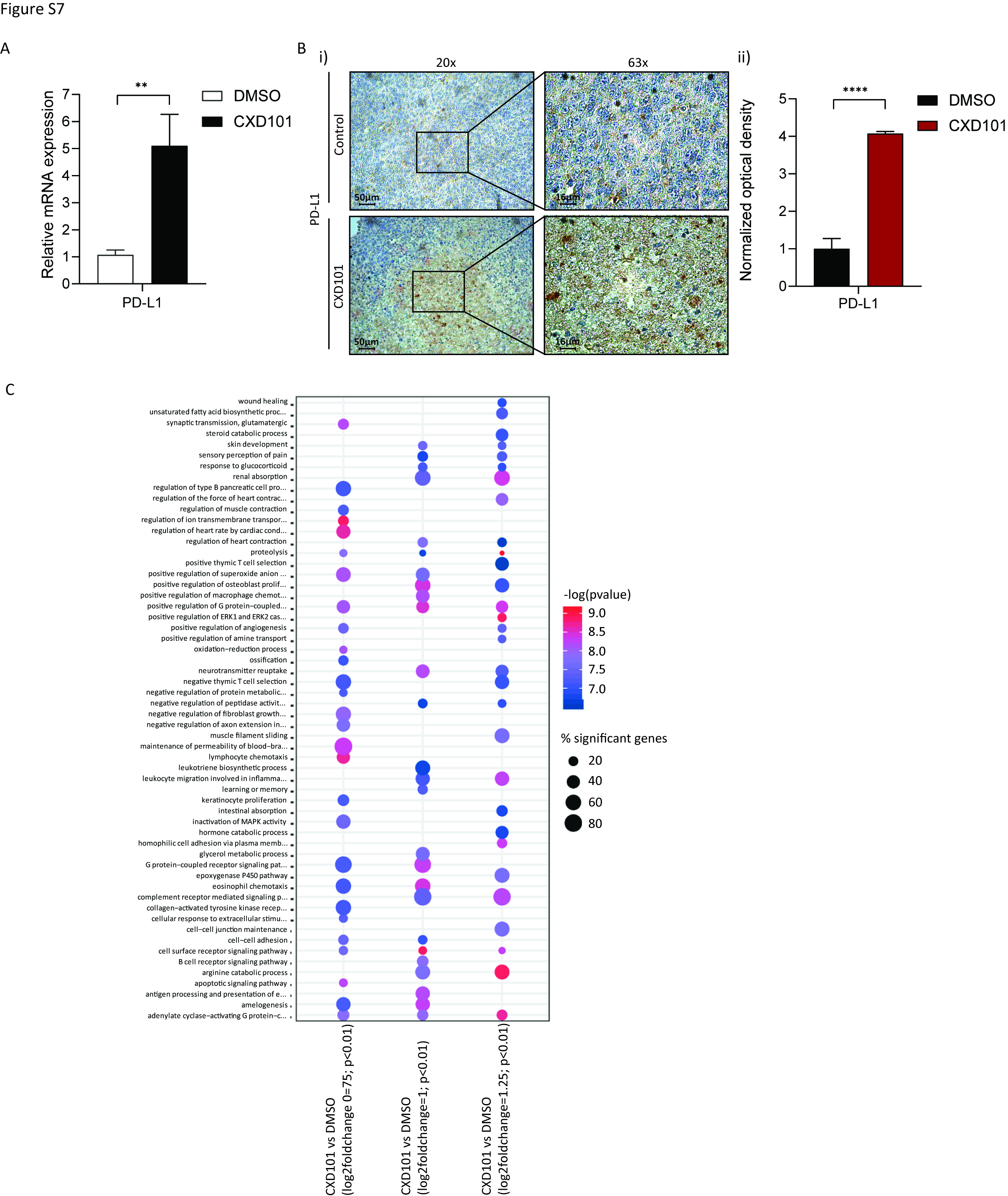

Supplement: Supplementary file 7 — Fig. S7. Effect of CXD101 on PD‐L1 expression in colon26 syngeneic mouse model and general gene ontology analysis. [file MOL2-15-3280-s006.tif]
