# Supplementary material for: Immune modulation underpins the anti‐cancer activity of HDAC inhibitors
Source: Mol Oncol. 2021 May 1;15(12):3280–98. doi: 10.1002/1878-0261.12953 (PMC8637571; doi:10.1002/1878-0261.12953)
Supplement: Supplementary file 11 — Supplementary Material [file MOL2-15-3280-s003.docx]

**Supplementary data**

**Immune modulation underpins the anti-cancer activity of HDAC inhibitors**

Wiktoria Blaszczak^1∞^, Geng Liu^2∞^, Hong Zhu^2,3^, Wojciech Barczak^2^, Amit Shrestha^2^, Gulsah Albayrak^2^, Shunsheng Zheng^1^, David Kerr^1,4^, Anastasia Samsonova^5,6^ and Nicholas B La Thangue^1,2,*^

^1^ Celleron Therapeutics Ltd, Magdalen Centre, Oxford Science Park,

Oxford, OX4 4GA, United Kingdom

^2^ Laboratory of Cancer Biology, Department of Oncology, University of Oxford

Old Road Campus Research Building, Oxford, OX3 7DQ, United Kingdom

^3^ Department of Medical Oncology, Cancer Center, West China Hospital, Sichuan University, Chengdu, 610041, China

^4^ Nuffield Division of Clinical Laboratory Sciences, Academic Block, University of Oxford, Oxford, United Kingdom.

^5^ Centre for Computational Biology, Peter the Great Saint Petersburg Polytechnic University, St. Petersburg 195251, Russia

^6^ Centre for Genome Bioinformatics, St. Petersburg State University, St. Petersburg 199034, Russia

∞ Each author made an equal contribution

* Corresponding Author

Running title: HDAC inhibitors regulate immune recognition

Keywords: HDAC inhibitors, tumour microenvironment, immunotherapy, checkpoints inhibitors

**Supplementary Figure 1: Effect of CXD101 on SW620 and colon26 colon cancer cells. A)** (i) A representative immunoblot demonstrating H3AcK14 levels in SW620 cells after 2 days of treatment with indicated concentrations of CXD101; histone H3 wasincluded as a loading control; (ii) quantification of immunoblots by ImageJ software. Normalised optical density was presented as a mean +/- SD, n=3 (Student’s t-test; *p < 0.05). **B)** IC50 plots from MTT assays performed after treating SW620 cells for 72 and 120 hours with increasing concentrations of CXD101. **C)** (i) A representative immunoblot demonstrating H3AcK9 levels in colon26 cells after 3 days of treatment with the two concentrations of CXD101; histone H3 included as a loading control; (ii) quantification of immunoblots by ImageJ Fiji software. Normalised optical density was presented as a mean +/- SD, n=3 (Student’s t-test; *p < 0.05). **D)** IC50 plots from MTT assays performed after treating colon26 cells for 3 and 5 days with increasing concentrations of CXD101.

**Supplementary Figure 2. Effect of CXD101 on genes within the AP and NK signature in SW620, MCF7, A549, and HCT116 cells. A)** Graphical representation of normalised rlog-transformed gene expression values showing significantly differentially expressed genes in CXD101-treated (n=2) and control (n=3) SW620 cells, associated with Reactome immune system (AP signature) (i) and “Natural Killer Cell Mediated Cytotoxicity” KEGG pathway (NK signature) (ii). **B)** Graphical representation of the ‘antigen processing and presentation’ (AP) genes in the MCF7 cell data set (27) generated using the Genevestigator tool, presented as log2(FPKM) expression level. **C)** Quantitative reverse transcription PCR (qRT-PCR) of MHC-related genes within the AP signature (Fig. 1D) in MCF7 cells treated for 3 days with CXD101 (5 µM) or DMSO control (Student’s t-test; *p < 0.05, error bars indicate SD); immunoblot of MCF7 cell extracts showing H3AcK14; n=3. **D)** Graphical representation of the ‘antigen processing and presentation’ (AP) genes in the A549 cell data set (27) generated using the Genevestigator tool, presented as log2(FPKM) expression level. **E)** Quantitative reverse transcription PCR (qRT-PCR) of MHC-related genes in the AP signature in A549 cells treated for 3 days with CXD101 (5 µM) or DMSO control (Student’s t-test; *p < 0.05, error bars indicate SD); immunoblot of A549 extracts to demonstrate levels of H3AcK14; n=3. **F)** Graphical representation of the ‘antigen processing and presentation’ (AP) genes in the HCT116 cell data set (27) generated using the Genevestigator tool, presented as log2(FPKM) expression level. **G)** Quantitative reverse transcription PCR (qRT-PCR) of MHC-related genes in the AP signature in HCT116 cells treated for 3 days with CXD101 (5 µM) or DMSO control (Student’s t-test; *p < 0.05, error bars indicate SD); immunoblot to demonstrate input protein levels for H3AcK14; n=3.

**Supplementary Figure 3. Effect of CXD101 on genes in the AP and NK signatures in colon26 cells *in vitro* and *in vivo*. A)** Graphical representation of normalised rlog- transformed gene expression values showing significantly differentially expressed genes in CXD101-treated colon26 cells (n=3) and control colon26 cells (n=3), associated with Reactome immune system (AP) (i) and “Natural Killer Cell Mediated Cytotoxicity” KEGG pathway (NK). **B)** Graphical representation of normalised rlog-transformed gene expression values showing significantly differentially expressed genes in CXD101-treated and control colon26 tumours (n=3), associated with Reactome immune system (AP) (i) and “Natural Killer Cell Mediated Cytotoxicity” KEGG pathway (NK).

**Supplementary Figure 4. Treatment with CXD101 and anti-CTLA4 in colon26 tumours.** **A)** Schematic representation of experiment. Balb/c mice were treated with orally administrated CXD101 (50 mg/kg; five-days-on/two-days-off schedule) for 38 days or with vehicle only control. Group 3 was treated with anti-mCTLA4 administered intraperitoneally on day 1 (5 mg/kg) and days 3 and 6 (2.5 mg/kg). Group 4 received a combination of CXD101 with anti mCTLA4; n=6. **B)** Scatter plots of relative tumour volume of individual mouse at day 15 (Student’s t-test; *p < 0.05) **C)** Relative tumour growth analysis of treated and control mice presented as a mean value (Student’s t-test; *p < 0.05). **D)** Relative body weight of treated and non-treated mice presented as a mean value **E)** survival curves of treated and non-treated mice (Log-rank (Mantel-Cox) test; *p < 0.05)**.**

**Supplementary Figure 5. Comparison with other HDAC inhibitors and disease spectrum. A)** Heatmap representation of the (AP) and (NK) signatures in PC-3 (ii and iii) and NCI-H295R (iv) cell data sets treated with the indicated HDAC inhibitors generated using the Genevestigator tool, presented as normalised expression level using Morpheus software. A heatmap from the SW620 qPCR analysis (Fig 1F) was included for comparison (i); n=3. **B)** Heatmap representation of expression levels for AP and NK signatures in human cancer compared with normal tissue, generated using Morpheus software. Data from The Cancer Genome Atlas (TCGA) and Genotype-Tissue Expression projects collected from Xena Browser were used to display expression levels from cancer tissue or healthy tissue. Data are presented as a normalised mean expression level. For detailed analysis of microsatellite stability and staging, TCGA colon, stomach, and oesophageal cancer data sets collected from cBioportal were used. Data are presented as a normalised mean expression level. In the staging analysis, high expression levels are indicated with darker red colouring, whereas low expression levels are indicated with white colouring (i). In microsatellite stability analysis, high expression levels are indicated with green colouring, whereas low expression levels are indicated with white colouring (ii).

**Supplementary Figure 6. Comparison with other HDAC inhibitors and disease spectrum. A)** Quantification of genes in the AP (i) and NK (ii) signatures in PC-3 cells treated with entinostat. Results are expressed as average (mean) fold change compared to control treatments. Error bars represent SD unless otherwise indicated (Student’s t-test; *p < 0.05). **B)** Quantification of the AP (i) and NK (ii) signatures in PC-3 cells treated with vorinostat. Results are expressed as average (mean) fold change compared to control treatments. Error bars represent SD unless otherwise indicated (Student’s t-test; *p < 0.05). **C)** Quantification of the AP (i) and NK (ii) signatures in NCI-H295R cells treated with panobinostat. Results were expressed as average (mean) fold change compared to control treatments. Error bars represent SD unless otherwise indicated (Student’s t-test; *p < 0.05). **D)** Quantification of the AP (i) and NK (ii) signatures in MOLM-13 cells treated with chidamide. Results were expressed as average (mean) fold change compared to control treatments.

**Supplementary Figure 7. Effect of CXD101 on PD-L1 expression in colon26 syngeneic mouse model and general gene ontology analysis. A)** Quantitative reverse transcription PCR (qRT-PCR) validation of PD-L1 gene in colon26 syngeneic tumour RNA treated for 14 days with 50 mg/kg CXD101 or DMSO control; n=3 (Student’s t-test; *p < 0.05; error bars indicate SD). **B)** Representative examples (i) of immunohistochemical staining of PD-L1 in colon26 tumours collected from Balb/c mice at 14 days treated with 50 mg/kg CXD101 and non-treated control (see experiment in figure 3A). Original magnification: 20x, scale bar, 50 μm; and 63x; scale bar, 16 μm. n=4; (ii) Results were quantified by ImageJ Fiji software and normalised optical density was presented as a mean +/- SD. Statistical analysis was performed using two-tailed, unpaired Student’s t-test with GraphPad Prism 8 software, n=4. **C)** Gene Ontology (GO) biological process enrichment analysis on from the colon26 RNA-seq data. GO biological process enrichment analysis was performed with statistically significant (p value<0.01) upregulated differentially expressed genes (fold change over 0.75, 1, and 1.25 log2 fold change).

**Supplementary datasets:**

**Supplementary dataset 1.** List of up- and down-regulated genes after HDAC inhibition in SW620 cells identified from the RNA-seq analysis.

**Supplementary dataset 2.** List of up- and down-regulated genes after HDAC inhibition in colon26 cells identified from the RNA-seq analysis.

**Supplementary dataset 3.** List of up- and down-regulated genes after HDAC inhibition in colon26 tumours *in vivo* identified from the RNA-seq analysis.
